# Polygenic Variants Linked to Oxidative Stress and the Antioxidant System Are Associated with Type 2 Diabetes Risk and Interact with Lifestyle Factors

**DOI:** 10.3390/antiox12061280

**Published:** 2023-06-15

**Authors:** Youngjin Choi, Hyuk-Ku Kwon, Sunmin Park

**Affiliations:** 1 Department of Food Science & Technology, Hoseo University, Asan 31499, Republic of Korea; ojchoi@hoseo.edu; 2 Department of Environmental Engineering, Hoseo University, Asan 31499, Republic of Korea; hkkwon@hoseo.edu; 3 Department of Food and Nutrition, Obesity/Diabetes Research Center, Hoseo University, Asan 31499, Republic of Korea

**Keywords:** type 2 diabetes, oxidative stress, antioxidants, vitamin D, bioactive compounds, coffee

## Abstract

Oxidative stress is associated with insulin resistance and secretion, and antioxidant systems are essential for preventing and managing type 2 diabetes (T2DM). This study aimed to explore the polygenic variants linked to oxidative stress and the antioxidant system among those associated with T2DM and the interaction of their polygenic risk scores (PRSs) with lifestyle factors in a large hospital-based cohort (*n* = 58,701). Genotyping, anthropometric, biochemical, and dietary assessments were conducted for all participants with an average body mass index of 23.9 kg/m^2^. Genetic variants associated with T2DM were searched through genome-wide association studies in participants with T2DM (*n* = 5383) and without T2DM (*n* = 53,318). The Gene Ontology database was searched for the antioxidant systems and oxidative stress-related genes among the genetic variants associated with T2DM risk, and the PRS was generated by summing the risk alleles of selected ones. Gene expression according to the genetic variant alleles was determined on the FUMA website. Food components with low binding energy to the GSTA5 protein generated from the wildtype and mutated *GSTA5*_*rs7739421* (missense mutation) genes were selected using in silico analysis. Glutathione metabolism-related genes, including glutathione peroxidase (*GPX)1* and *GPX3*, glutathione disulfide reductase (*GSR)*, peroxiredoxin-6 (*PRDX6)*, glutamate–cysteine ligase catalytic subunit (*GCLC)*, glutathione S-transferase alpha-5 (*GSTA5)*, and gamma-glutamyltransferase-1 (*GGT1*), were predominantly selected with a relevance score of >7. The PRS related to the antioxidant system was positively associated with T2DM (ORs = 1.423, 95% CI = 1.22–1.66). The active site of the GASTA proteins having valine or leucine at 55 due to the missense mutation (*rs7739421)* had a low binding energy (<−10 kcal/mol) similarly or differently to some flavonoids and anthocyanins. The PRS interacted with the intake of bioactive components (specifically dietary antioxidants, vitamin C, vitamin D, and coffee) and smoking status (*p* < 0.05). In conclusion, individuals with a higher PRS related to the antioxidant system may have an increased risk of T2DM, and there is a potential indication that exogenous antioxidant intake may alleviate this risk, providing insights for personalized strategies in T2DM prevention.

## 1. Introduction

Type 2 diabetes (T2DM) is a metabolic disorder characterized by high blood sugar levels due to increased insulin resistance and relative insulin deficiency [1]. It typically develops in adulthood and is most often diagnosed in adults over the age of 45. The prevalence of T2DM in Asia is high and increasing. According to the World Health Organization (WHO), the number of persons with T2DM was about 415 million in 2019 in Asia [2] and 538 million in 2021 worldwide [3]. This number is expected to reach 642 million by 2045 [2]. Specifically, countries in south and east Asia have some of the highest T2DM prevalence rates in the world [4].

The etiology of T2DM is insulin resistance, primary β-cell failure, or their sequential or simultaneous combination linked to oxidative stress [5]. High levels of glucose and free fatty acids in the circulation exacerbate oxidative stress-induced β-cell dysfunction, a decrease in proliferation, and an increase in β-cell apoptosis. Indeed, the pancreatic β-cells are highly susceptible to oxidative stress due to a decrease in the enzymes associated with the antioxidant defense system, such as superoxide dismutase (*SOD*), glutathione peroxidase (*GPX*), and catalase; an increase in reactive oxygen species (ROS) and reactive nitrogen species results in β-cell dysfunction and reduced β-cell mass [5]. Oxidative stress in the peripheral tissues also plays a role in inducing insulin resistance [6,7]. Emerging data from epidemiological studies indicate that Asians have a lower insulin-secreting capacity than Caucasians [8,9]. Therefore, in Asians who are challenged with unhealthy lifestyles, in addition to factors such as aging, obesity, inflammation, and oxidative stress, which increase insulin resistance, as well as decrease β-cell mass and insulin secretion, progression to T2DM occurs more quickly compared to Caucasians [10,11].

It is well established that T2DM is highly correlated with oxidative stress. Pro-oxidants and biomarkers of oxidative stress-induced tissue damage, oxidized DNA bases, hydroperoxides, 8-hydroxy-deoxyguanine, and 8-epi-prostaglandin F2α are elevated in the pancreas, white blood cells, and plasma of patients with T2DM [12]. The total antioxidant status significantly decreases in T2DM. It is associated with decreased levels of antioxidant enzymatic (such as GPX, catalase, and SOD) and nonenzymatic (such as vitamins C and E and glutathione) components [13]. T2DM patients exhibit low levels of reduced glutathione and high levels of oxidized glutathione (GSSG) [14]. The increased oxidative stress in T2DM leads to inflammation and cell and tissue damage, contributing to an increased risk of high blood pressure, cardiovascular diseases (including myocardial infarction, atherosclerosis, and stroke), and other serious health problems [15,16]. Therefore, oxidative stress is strongly correlated with the pathogenesis of T2DM and its complications.

Earlier studies suggested that oxidative stress is linked to genetic and environmental factors, and their interaction results in the development of T2DM and its complications. The oxidative stress-related genetic variants of *GPX1*, *GPX3*, glutathione S-transferase (*GST*)-theta-1 *(GSTT1)*, GST-mu1 *(GSTM1)*, arachidonate 5-lipoxygenase *(ALOX5),* and cytochrome β-245 alpha chain *(CYBA)* have been found to influence T2DM risk [6,17,18]. However, these studies were conducted in small groups of participants, and genetic variants related to oxidative stress and antioxidant systems have yet to be studied in larger populations. Moreover, the interaction between these genetic variants and lifestyle factors has yet to be examined. The present study aimed to examine the hypothesis that the polygenic variants related to oxidative stress and the antioxidant system were associated with T2DM, and that their polygenic risk score (PRS) interacted with lifestyle factors, including nutrient and polyphenol intake, in a large hospital-based cohort (*n* = 58,701). The present study is novel in revealing that the PRS of the antioxidant system is positively associated with T2DM risk, and it interacts with dietary antioxidants such as bioactive compounds, vitamins C and D, and coffee intake. Therefore, personalized nutrition can be designed according to the PRS.

## 2. Materials and Methods

### 2.1. Data Acquisition

The institutional review board (IRB) of the Korea National Institute of Health approved the Korean Genome and Epidemiology Study (KoGES; KBP-2015-055). All participants signed a written informed consent form. Participants over 40 years who had volunteered in a large city hospital-based cohort as part of the KoGES conducted from 2010 to 2014 were included in the study [19]. The participants’ demographic, anthropometric, biochemical, dietary, and genetic data were collected under a well-controlled system. The data were stored in the National Biobank of Korea (Osong, Republic of Korea). They were obtained for the study after receiving IRB approval from Hoseo University (HR-034-01).

The previous studies showed anthropometric and biochemical measurements using a standard method in the KoGES [19,20,21]. Insulin resistance was determined using the Homeostatic Model Assessment for Insulin Resistance (HOMA-IR) and predicted using the XGBoost model generated with the Ansung/Ansan cohort [22]. The cutoff for insulin resistance was 2.32 [22,23]. The data were modulated for the analysis, such as defining and categorizing education, alcohol intake, smoking status, and exercise [20,21]. Food intake was determined using a semi-quantitative food frequency questionnaire (SQFFQ) generated to reflect the Korean diet, suitable for measuring food intake in large cohort studies. Dietary patterns were categorized into the Korean balanced diet (KBD), plant-based diet (PBD), Western-style diet (WSD), or rice-based diet (RBD) using principal component analysis (PCA) with 30 predefined groups from 106 food items in the SQFFQ. The conditions for the PCA were the use of the orthogonal rotation procedure (varimax) and eigenvalues >1.5. The names of the dietary patterns were assigned to food groups with ≥0.40 factor loading values, which were considered the predominant contributors.

The dietary inflammatory index (DII), glycemic index (GI), and glycemic load (GL) of the daily meal and sulfur microbial diet were calculated with the designated equations [21,24]. DII, an index of the inflammatory potential of dietary components, was calculated by multiplying the dietary inflammatory scores of the 38 food and nutrient components by their daily intakes. The sum of the scores of the above items was divided by 100, as described previously. The GI was calculated by summing the values obtained by multiplying the GI for each food item by its amount. The GI values of food items commonly consumed by Koreans were used [25]. The sulfur microbial diet scores were calculated by summing the result of the multiplication of the beta coefficients with the food amounts [26]. Food coefficients were assigned on the basis of the growth of 43 gut microbes, as in an earlier study [26]. The food groups positively associated with sulfur-metabolizing bacteria growth were processed meats, liquor, and low-calorie drinks. Those negatively associated with growth were beer, fruit juice, legumes, other vegetables, and sweets or desserts. Regular exercise was defined as over 150 min per week of moderate-intensity physical activity such as brisk walking, water aerobics, bike riding, dancing, doubles tennis, pushing a lawn mower, hiking, and rollerblading.

### 2.2. Genotyping Using a Korean Chip and Quality Control

The genotyping of the genomic DNA isolated from the blood of the participants (*n* = 58,701) was conducted using a customized K-chip containing 833,000 variants (Axiom Biobank plus Genotyping Array, KNIHv1.1), which contained a tagging SNP that maximized genomic coverage, as well as functional SNPs such as the nonsynonymous, quantitative expression trait loci (eQTL), and previously reported disease-associated SNPs. Reproducibility and accuracy of the K-chip were reported to be 99.77% and 99.73%, respectively (Affymetrix, Santa Clara, CA, USA), designed to examine the disease-related single-nucleotide polymorphisms (SNPs) in Asians at the Center for Genome Science at the Korea National Institute of Health. The genotypes were imputed with the 1000 Genomes sequence or the data from the Korean HapMap Project [27]. The incorrect genetic variants were excluded on the basis of the following criteria: missing genotype call rate ≥4%, heterozygosity >30%, genotyping accuracy <98%, gender bias, minor allele frequency (MAF) <1%, and the Hardy–Weinberg equilibrium (HWE) *p* < 0.05 [28].

### 2.3. Genetic Variants Associated with T2DM

T2DM was defined as a fasting plasma glucose level ≥126 mg/dL or HbA1c ≥6.5%. Additionally, the T2DM group included participants who were currently taking antidiabetic medications. The number of T2DM and non-T2DM participants was 5383 and 53,318, respectively. The genetic variants for T2DM were identified through a genome-wide association study (GWAS), and their appropriateness was checked with Manhattan and quantile–quantile (QQ) plots. The lambda value of the QQ plot was close to 1, and the genotypes by GWAS were appropriate. Genetic variants having D′ < 0.2 in the linkage disequilibrium (LD) criteria were selected using Haploview 4.2 in PLINK. Oxidative stress-related genes (*n* = 211; Supplementary Table S1) were obtained from Gene Cards (https://www.genecards.org, accessed on 7 June 2022) with a relevance score ≥7.

### 2.4. Gene Ontology (GO) and Kyoto Encyclopedia of Gene and Genomes (KEGG) Pathway Enrichment Analysis

Among the genetic variants associated with T2DM, those associated with oxidative stress-related genes were selected. GO enrichment and KEGG pathway analyses were conducted to discover the biological functions of selected data acquisition and differentially expressed oxidative stress genes (DEOSGs) using the Database for Annotation, Visualization, and Integrated Discovery (DAVID) version 6.8 (https://david.ncifcrf.gov/tools.jsp, accessed on 13 July 2022). The genes related to oxidative stress were selected with the criterion of a relevance score >7, indicating that the selected genes were associated with oxidative stress, which was categorized into the response to oxidative stress and the antioxidant system. The response to oxidative stress is a cellular or biological process that occurs in response to an increase in oxidative stress levels and involves various cellular pathways and molecular mechanisms to mitigate the damage caused by ROS [29]. The antioxidant system includes enzymes such as SOD, catalase, GPX, and various nonenzymatic antioxidants such as glutathione and vitamins C and E, as well as the response to oxidative stress and the antioxidant system [29]. These components act cooperatively to scavenge ROS. Therefore, the antioxidant system is part of the response to oxidative stress. The statistical significance of the *p* and false discovery rate (FDR) values was assigned as 0.05 [30].

### 2.5. PRS Generation

PRSs for the response to oxidative stress and the antioxidant system were calculated by summing the number of the risk alleles from each selected SNP in the allelic genetic model [31,32,33]. The PRSs indicated the genetic risk for T2DM associated with oxidative stress or the antioxidant system. The PRS for the response to oxidative stress was divided into three categories: low PRS (<8), medium PRS (8–9), and high PRS (>9). The PRS values for the antioxidant system were divided into three categories: low PRS (<9), medium PRS (9–11), and high PRS (>11). The high PRS group had the highest genetic risk for T2DM linked to oxidative stress or antioxidant system. The PRS categories enabled a more detailed understanding of the interplay between genetic variants related to oxidative stress and the antioxidant defense system in the development of T2DM. The PRS was calculated in dominant and recessive genetic models. Heterozygotes were considered a risk group in the dominant genetic model, while they were a non-risk group in the recessive genetic model.

### 2.6. Genotype-Tissue Expression (GTEx) of Genetic Variants and Their Distribution of Tissue/Organs

The GTEx of genetic variants was found using the source code of the functional mapping and annotation of the genetic association (FUMA) web application. The normalized gene expression with reads per kilobase of transcript per million reads mapped (RPKM) was obtained in the 53 tissue types from the GTEx portal. A total of 56,320 genes were accessible in the GTEx dataset, and we applied a filter to include only those genes present in tissues with an average RPKM value greater than or equal to 1 in at least one tissue type.

### 2.7. Molecular Docking of the Genes Having a Missense Mutation with Food Compounds and Molecular Dynamics Simulation (MDS)

The protein structure containing the missense mutation was obtained in Protein Data Bank (PDB) format from the Iterative Threading Assembly Refinement (I-TASSER) website (https://zhanggroup.org/I-TASSER/, accessed on 17 October 2022). Subsequently, it was converted into a Protein Data Bank, partial charge, and atom type (PDBQT) format using AutoDock Tools 1.5.6 (Molecular Graphics Laboratory, Scripps Research Institute, FL, USA) [34]. The active sites of the protein were searched on the ProteinsPlus website (https://proteins.plus/, accessed on 30 October 2022), and the active functional pockets and the mutated sites were also included in the active site for molecular docking. Food compounds (*n* = 20,000) in the PDBQT format bound to the active site of the protein structure file in the PDBQT format were selected in the Vena program [34]. Food components having <−10 kcal/mol binding energy with the protein PDBQT were selected [35]. The results indicated that a lower binding free energy resulted in a tighter binding affinity. The molecular docking and gene expression results can provide additional support for how the genetic variants affect T2DM etiology.

The conformational changes in the protein structures were examined using MDS to detect the changes in their activity. The Chemistry at Harvard Macromolecular Mechanics (CHARMM) force field was added to the docked complex in the “Simulation” part, and the protein was solvated by “Solvation”. The molecular dynamics simulation parameters for the protein in the solvent system were established using the “Standard Dynamics Cascade” method. After a 10 ns simulation, the root-mean-square deviation (RMSD), root-mean-square fluctuation (RMSF), and hydrogen bond values were determined to assess the protein’s conformational changes and stability.

### 2.8. Statistical Analysis

The sample size was 27,853 participants with the condition of α = 0.05, β = 0.99, an odds ratio (ORs) of 1.05, and about 10% of prevalence in the logistic analysis using a G-power calculator. The total size of 58,701 participants was sufficient for the study. After adjusting for the covariates, the adjusted means with standard errors were calculated for the continuous variables. Their statistical differences were evaluated using a two-way analysis of covariance (ANCOVA) [36]. Multiple comparisons among the groups were assessed using Tukey’s test to determine if the ANCOVA was significant. Descriptive statistics for the categorical variables were assessed using frequency distributions with the chi-square test.

The adjusted ORs and 95% confidence intervals (CIs) for the PRS and T2DM were determined using logistic regression analysis after adjustment for covariates. Two covariate sets were used for the logistic regression analysis. Covariate set 1 included age, residence area, survey year, body mass index (BMI), education, and income as covariates. Covariate set 2 was calculated using covariate set 1 plus energy intake, physical activity, smoking status, and alcohol and coffee consumption.

The interaction between the PRS and lifestyle parameters for T2DM was evaluated with a two-way ANCOVA containing an interaction term. When the interaction term was statistically significantly different, the adjusted ORs and 95% CIs for the PRS and T2DM were calculated using logistic regression after adjusting for covariate set 2 in the low and high groups of each lifestyle. The cutoffs for each lifestyle factor were determined on the basis of either the dietary reference intake (DRI) or the 30th percentiles of each variable. The χ^2^ test was employed within the low and high groups of the lifestyle-related parameters according to the PRS categories.

## 3. Results

### 3.1. Characteristics of the Participants

The participants in the T2DM group included older individuals and more men than those without T2DM (Table 1). T2DM incidence was significantly different with education level according to the chi-square test (Table 1). The average BMI and waist circumference of the participants in the T2DM group were higher than in the participants without T2DM. The T2DM subjects exhibited much higher fasting plasma glucose and HbA1c concentrations and insulin resistance determined by HOMA-IR (Table 1; *p* < 0.001). The incidence of cardiovascular diseases, including myocardial infarction and stroke, was higher in the T2DM group (*p* < 0.001).

The energy and fat intakes were lower among T2DM subjects than non-T2DM subjects. There was no significant difference in the KBD and PBD intake between the T2DM and non-T2DM subjects, and the T2DM patients consumed less WSD and RMD than non-T2DM subjects. DII, GI, and GL were lower in the T2DM than the non-T2DM groups (*p* < 0.001), although they were within the acceptable ranges. However, the intake of bioactive compounds, including quercetin, luteolin, genistein, daidzein, and cyanidin (*p* < 0.01) and vitamin C (*p* < 0.05) was lower in the T2DM than in the non-T2DM group (Table 1).

There was no difference in alcohol intake between the two groups, and the incidence of regular exercise was lower in the T2DM than in the non-T2DM group. The number of former and current smokers was higher in the T2DM group than in the non-T2DM group. These results suggested that the T2DM patients were potentially more likely to have higher oxidative stress since they had a lower intake of antioxidants.

### 3.2. DEOSGs Identification and Functional Enrichment Analysis

Genetic variants associated with T2DM in GWAS were extracted for the 211 genes related to oxidative stress (>7 relevance score), and 11 genes with genetic variants significantly associated with T2DM were identified (Supplementary Table S1). The relevance scores of the 11 genes and their associations with T2DM, oxidative stress, and the antioxidant system are shown in Table 2: The relevance scores of T2DM and oxidative stress were over 7, but those of the antioxidant system ranged from 15.1 to 3.11.

**Figure 1:**
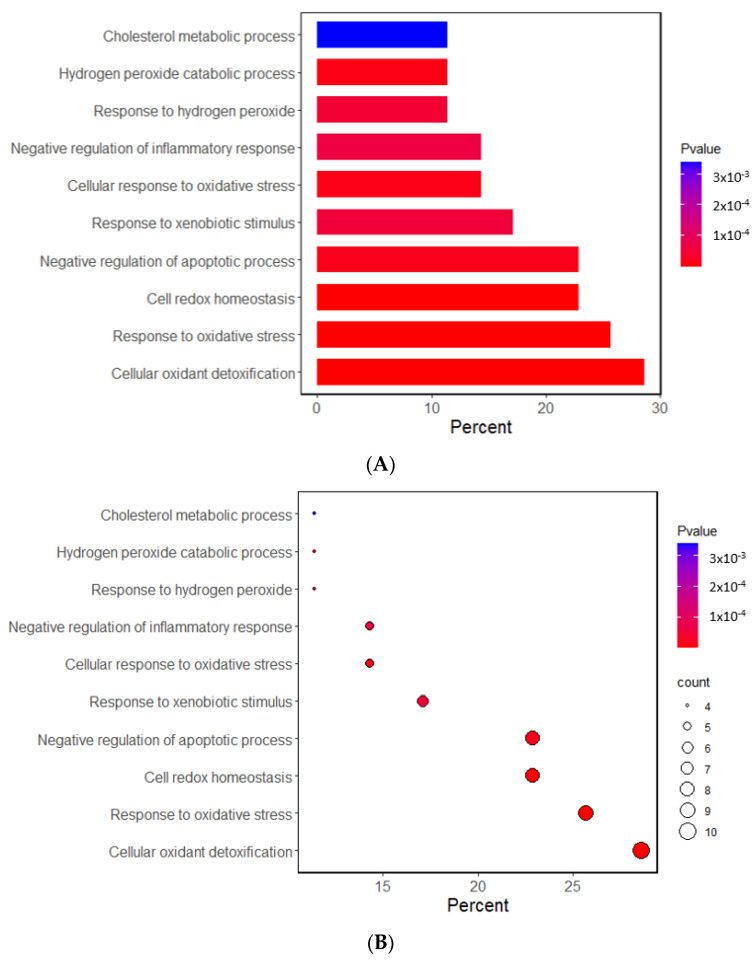

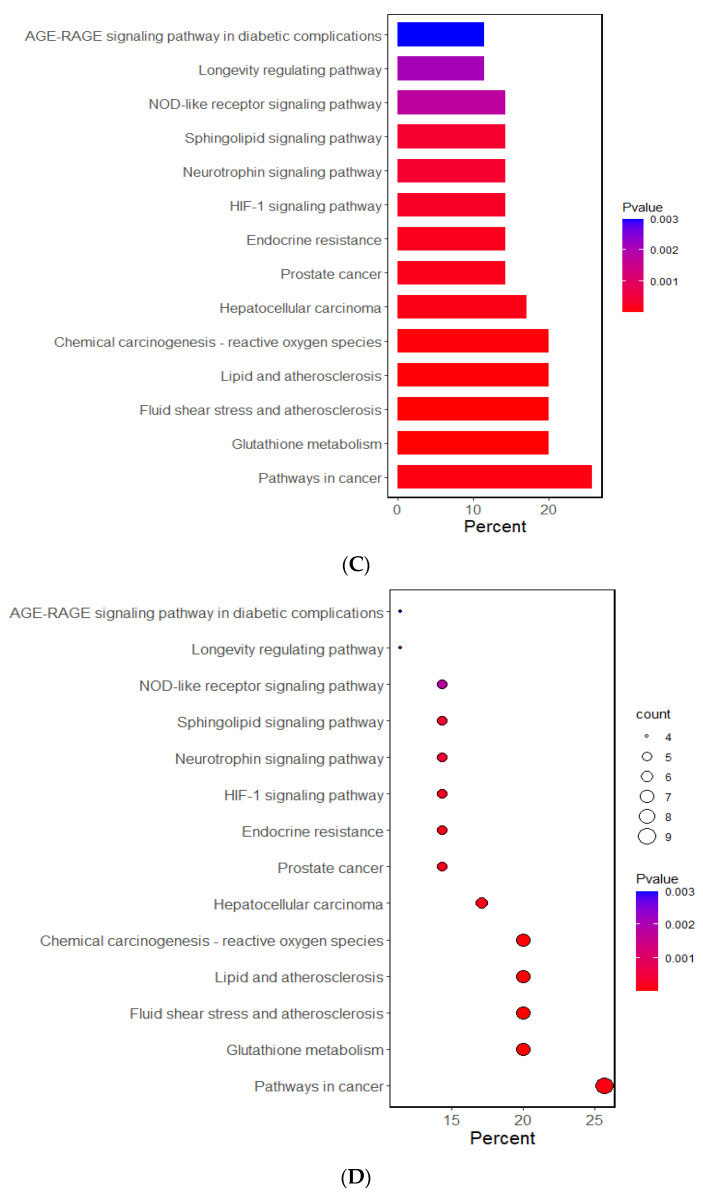
Functional enrichment analysis of the response to oxidative stress and antioxidant system-related genes. (**A**) The Gene Ontology (GO) term for the response to oxidative stress-related genes. (**B**) The Kyoto Encyclopedia of Genes and Genome terms (KEGG) for the response to oxidative stress-related genes. (**C**) The GO term for the antioxidant system-related genes. (**D**) The KEGG term for the antioxidant system-related genes.

The Gene Ontology (GO) and Kyoto Encyclopedia of Genes and Genome (KEGG) analyses investigated the potential functional and molecular mechanisms of these 11 genes linked to T2DM and oxidative stress. The pathways enriched with these genes are displayed in Figure 1. The GO analysis revealed that the biological process linked to oxidative stress was mainly linked to the selected genes. The GO revealed that 10 pathways were significantly associated with the selected genes by 11.4–28.6% in the relevant biological process of GO at the *p*-value of the Bonferroni correction (*p* < 0.05). The predominant pathways were cellular detoxification, cell redox homeostasis, response to oxidative stress, and negative regulation of apoptosis and inflammatory response (Figure 1A,B). In the KEGG analysis, the genes were associated with 14 pathways, including cancer, glutathione, atherosclerosis, lipid metabolism, and endocrine resistance (mainly insulin resistance) (Figure 1C,D). These results suggest that the genetic variants related to T2DM risk were linked to oxidative stress and the antioxidant system.

### 3.3. Genetic Variants Involved in the Antioxidant System and Response to Oxidative Stress Linked to T2DM Risk

**Figure 2:**
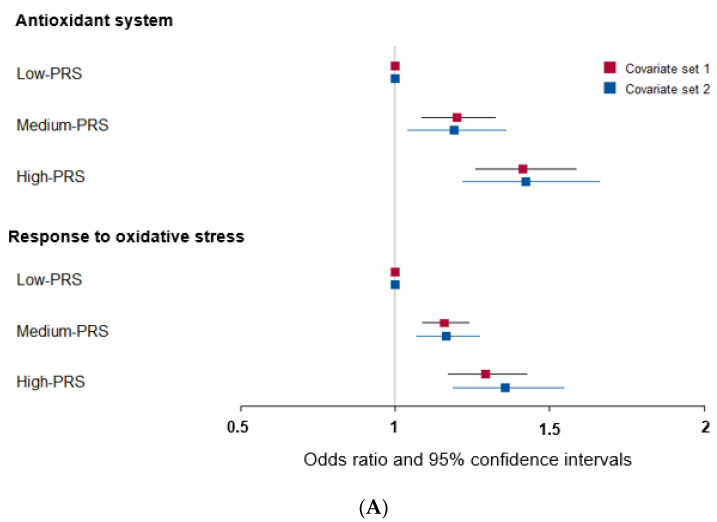

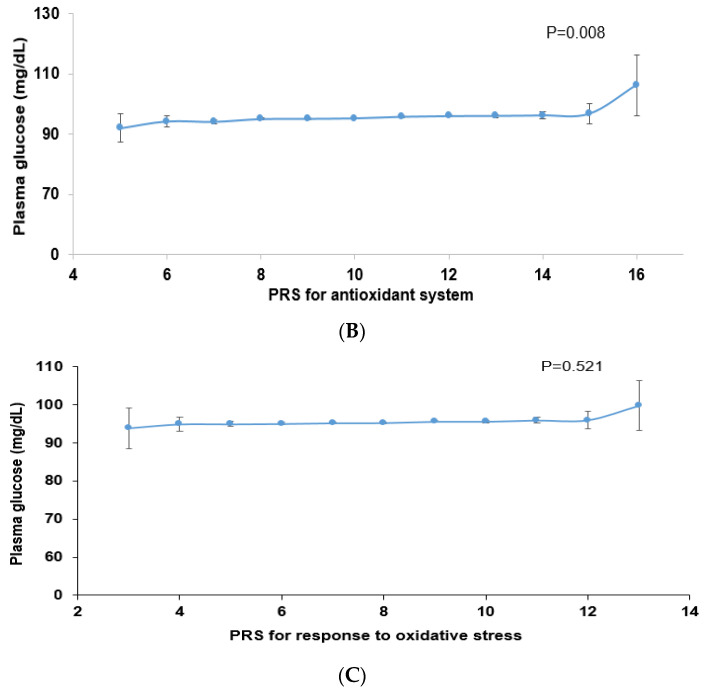
Association of polygenic risk scores (PRS) with type 2 diabetes risk (**A**) and fasting plasma glucose concentration (**B**,**C**) according to PRS in the allelic genetic model. The PRS models for the antioxidant system and the response to oxidative stress associated with type 2 diabetes risk. The PRS was calculated by summing the number of risk alleles of each SNP in the assigned model. The PRS for the antioxidant system was similarly categorized into <9, 9–11, and ≥12. The PRS for the response to oxidative stress was classified into the low, medium, and high groups, namely, <8, 8–9, and ≥10. In (A), the adjusted ORs for the PRS models were calculated by adjusting age, gender, education, income, occupation, residence area, and energy intake (percentage of estimated energy requirement) (covariate set 1), or variables in covariate set 1 plus regular exercise, alcohol intake, and smoking status (covariate set 2). Adjusted means and standard errors were calculated after adjusting for covariate set 2 in (**B**) and (**C**). Abbreviations: OR, odds ratio; PRS, polygenic risk score.

Among the genes related to the antioxidant system, genetic variants associated with T2DM were peroxiredoxin 6 *(PRDX6_rs150751487)*, *GPX1_rs1050614*, *GPX3_rs8177426*, glutathione S-transferase alpha 5 (*GSTA5)_rs7739421*, *GSTA5_rs2397118*, glutamate–cysteine ligase catalytic subunit *(GCLC)_rs74515451*, *GCLC_rs78386169*, glutathione disulfide reductase *(GSR)_rs10274638*, and gamma-glutamyltransferase 1 *(GGT1)_rs2076999*. Their genetic characteristics related to the antioxidant system are presented in Table 3A. Among them, *GSTA5_rs2397118* was a variant with a missense mutation. The wild and mutated types of *GSTA5* could have different binding affinities to bioactive components. Similarly, some genetic variants had a positive association, and others had a negative association with bioactive components. The PRS of the allelic genetic model linked to the antioxidant system was generated on the basis of the risk allele in each genetic variant. The PRS was positively associated with T2DM in the logistic regression model (ORs = 1.423, 95% CIs = 1.22–1.66) (Figure 2A). Furthermore, fasting plasma concentrations were significantly different among the PRS values (*p* = 0.008; Figure 2B). In a linear regression model, there was a significant positive linear association between PRS for antioxidant system and fasting plasma glucose concentration after adjusting for covariate set 2 (b = 0.34, *p* < 0.0001), indicating that, for each unit increase in PRS, there was a significantly estimated increase of 0.263 mg/dL in plasma glucose concentration. However, the PRS of the dominant and recessive genetic models linked to the antioxidant system showed a lower genetic impact on T2DM risk. Only the PRS of the recessive genetic model was significantly positively associated with T2DM after adjusting for covariate set 1, including age, gender, BMI, residence area, and education (ORs = 1.235, 95% CIs = (1.088–1.402) (Supplementary Figure S1).

Some genetic variants related to the response to oxidative stress were included in the antioxidant system, such as *PRDX6*_rs150751487, *GPX1*_rs1050614, *GPX3*_rs8177426, and *GCLC*_rs74515451, and other genetic variants such as selenoprotein P *(SELENOP)_rs28919269*, paraoxonase 2 *(PON2)_rs6462738*, and alleles of nuclear factor erythroid 2 *(NFE2)* including the basic-leucine zipper *(bZIP)* transcription factor 1 *(NFE2L1)_rs182345537*, and apolipoprotein E *(APOE)_rs769450* were included (Table 3). Their genetic characteristics related to the response to oxidative stress are presented in Table 3B. The PRS linked to the response to oxidative stress was positively associated with T2DM in a logistic regression analysis (OR = 1.354; 95% CI = 1.186–1.545) (Figure 2). However, fasting plasma concentrations did not significantly differ among the PRS values (*p* = 0.521). In a linear regression model, no significant linear association existed between PRS for cell response to oxidative stress and fasting plasma glucose concentration (b = 0.16, *p* = 0.053). However, the PRS of the dominant and recessive genetic models linked to the response to oxidative stress showed no genetic impact on T2DM risk (Supplementary Figure S1).

### 3.4. Gene Expression of GPX3 and GGT1 in Various Tissues according to Genetic Variants

**Figure 3:**
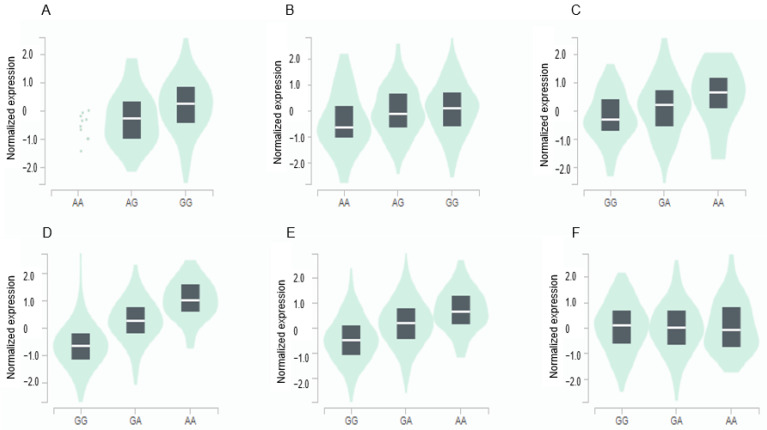
Genotype-Tissue Expression (GTEx) of genes according to their mutation. (**A**) *GPX3*_ rs8177426 in the cortex of the brain (β = 0.26, *p* = 0.0015). (**B**) *GPX3*_ rs8177426 in the left ventricle of the heart (β = 0.21, *p* = 9.1 × 10^−7^). (**C**) *GGT1*_ rs2076999 in the liver (β = 0.36, *p* = 2.7 × 10^−7^). (**D**) *GGT1*_ rs2076999 in the pancreas (β = 0.81, *p* = 1.4 × 10^−35^). (**E**) *GGT1*_ rs2076999 in the thyroid (β = 0.55, *p* = 8.8 × 10^−57^). (**F**) *GGT1*_ rs2076999 in the tibial nerve (β = −0.15, *p* = 0.00032). Abbreviations: *GPX*, glutathione peroxidase; *GGT1,* gamma-glutamyltransferase 1.

The *GPX3* expression was positively associated with the major allele (risk allele) of rs8177426 in the brain (β = 0.26, *p* = 0.0015) and heart (β = 0.21, *p* = 9.1 × 10^−7^) (Figure 3). The *GGT1* expression was positively related to minor allele (risk allele) of rs2076999 in the liver (β = 0.3, *p* = 2.7 × 10^−7^), pancreas (β = 0.81, *p* = 1.4 × 10^−35^), and thyroid (β = 0.55, *p* = 8.8 × 10^−57^) and inversely linked with it in the tibial nerve (β = −0.15, *p* = 0.00032) (Figure 3).

### 3.5. The Binding Energy of GSTA5_rs7739421 with Food Components

Since the missense mutation was observed only in the *GSTA5* gene, we conducted an in silico analysis to assess the functional implications of the wildtype and mutated versions of *GSTA5*_rs7739421. This analysis revealed how the mutated variants of *GSTA5*_rs7739421 may influence the potential function of the GSTA5 protein, specifically in relation to exogenous antioxidants, which can be applied to personalized nutrition. *GSTA5*_rs7739421 (missense mutation) has two alleles: C and T were converted from valine (Val; C) to leucine (Leu, T) at position 55, and T was the risk allele for T2DM. The active site of the GSTA5 proteins having Val or Leu at 55 had low binding energy (<−10 kcal/mol) with some bioactive components, particularly flavonoids and anthocyanins (Table 4). The proteins with Val or Leu at 55 exhibited the same bioactive compounds with similar or different binding energy. Asterlingulatoside D, kaempferol 3-O-rhamnosyl-rhamnosyl-glucoside, and (cyanidin 3-O-beta-glucoside)(kaempferol 3-O-(2-O-beta-glucosyl-beta-glucoside)-7-O-beta-glucosiduronic acid) malonate (CK-malonate) had low binding energy only with the active site of the GSTA5 protein having Leu at 55 (Table 4).

**Figure 4:**
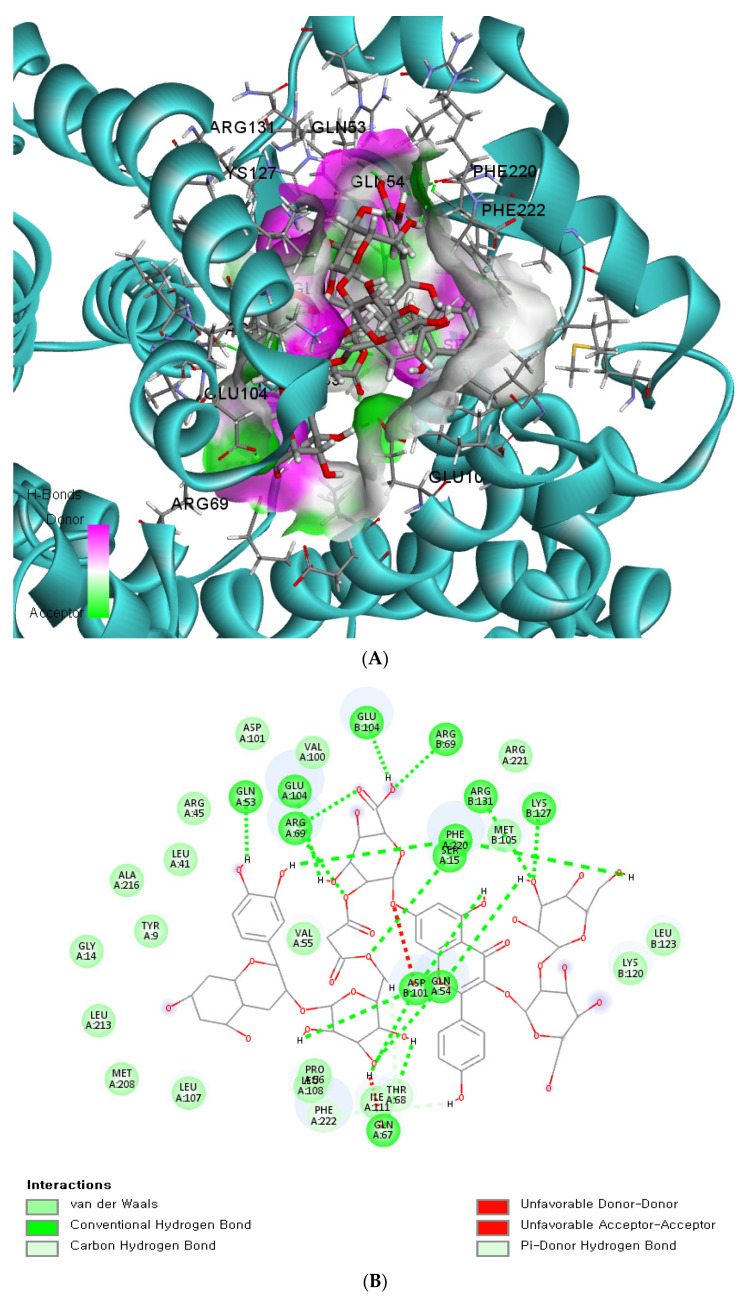

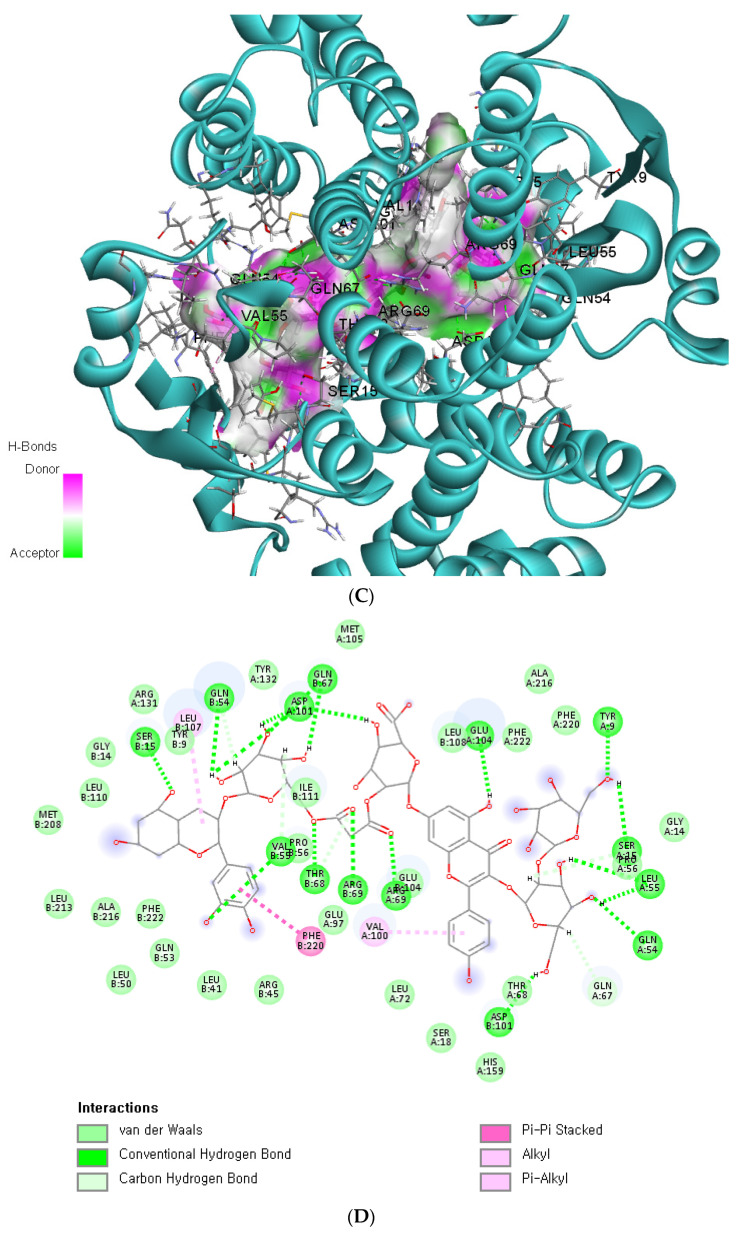

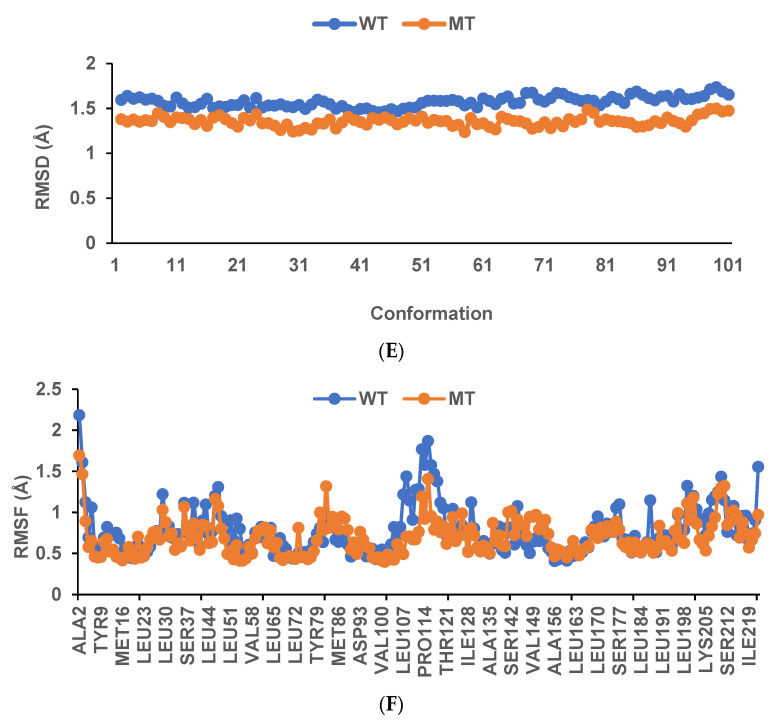
Molecular docking and molecular dynamic simulation (MDS) of CK-malonate on the wildtype (Val, C) of *GSTA5*_rs7739421(Val55Leu) and the mutated type (Leu, T). (**A**) Molecular docking of (cyanidin 3-O-beta-glucoside)(kaempferol 3-O-(2-O-beta-glucosyl-beta-glucoside)-7-O-beta-glucosiduronic acid) malonate (CK-malonate) to the *GSTA5*_rs7739421 wildtype. (**B**) The interaction force between CK-malonate and the *GSTA5*_rs7739421 wildtype. (**C**) Molecular docking of CK-malonate on the *GSTA5*_rs7739421 mutated type. (**D**) The interaction force between CK-malonate and the *GSTA5*_rs7739421 mutated type. (**E**). RMSD of CK-malonate on the *GSTA5*_rs7739421 wild and mutated types. (**F**) RMSF of CK-malonate on the *GSTA5*_rs7739421 wild and mutated types. Abbreviations: *GSTA5, glutathione S-transferase alpha 5*; RMSD, root- mean-square deviation; RMSF, root-mean-square fluctuation.

The binding affinity and interaction between CK-malonate and the wildtype (Val, C) *GSTA5*_rs7739421 are shown in Figure 4A,B. Figure 4C,D present the same for the mutated type (Leu, T) of *GSTA5*_rs7739421. The RMSD and RMSF for *GSTA5*_rs7739421 wild and mutated types binding to CK-malonate were further examined (Figure 4E,F). The RMSD for the mutated type binding with CK-malonate was observed to be lower than that for the wildtype; the mutated type sustained less than 1.5 Å for a duration of 100 ns, but the wildtype was higher than 1.5 Å. Likewise, the RMSF for most amino-acid residues of the mutated GSTA5 did not exceed 1.5 Å. However, the wildtype GSTA5 showed a higher RMSF than the mutated one. These collective results suggest that CK-malonate stably binds to the *GSTA5*_rs7739421 mutated type.

### 3.6. Interaction of the PRS Related to the Antioxidant System with Nutrient Intake

The PRS related to the antioxidant system was selected for further analysis since the linear regression of plasma glucose concentration was positively significantly different from the PRS of the antioxidant system (*p* < 0.0001) but not the PRS of the response to oxidative stress (*p* = 0.053). The antioxidant system was linked more to lifestyle factors than the response to oxidative stress. Since the PRS was generated from the genetic variants in the antioxidant system, its interaction was determined mainly with antioxidant-related food parameters such as PBD, DII, bioactive compounds (sum of quercetin, luteolin, genistein, daidzein, and cyanidin), vitamin D, coffee, and smoking status (Table 5). The PRS influenced the plasma glucose concentrations in the subjects with a high PBD intake compared to those with a low PBD intake (Table 5, Supplementary Figure S2A). In participants with an intake of foods with a high DII, the plasma glucose concentrations were positively associated with the PRS (Supplementary Figure S2B). However, the positive association between the PRS and DII was lower in those with a low-DII food intake than in those with a high-DII food intake (Table 5). The plasma glucose concentrations were lower in the low-DII than in the high-DII food intake subjects (Supplementary Figure S2B). Among the components of PBD, bioactive compounds, DII, vitamin C, vitamin D, and coffee intake showed an interaction with the PRS and influenced plasma glucose concentrations (Table 5). In subjects with a low bioactive compound intake, the plasma glucose concentrations were higher than in those with a high intake of bioactive compounds. They were not associated with the PRS in subjects with a high intake of bioactive compounds (Supplementary Figure S2C). In subjects with a low vitamin C and D intake, the plasma glucose concentrations were higher than in those with a high intake, and they were not significantly associated with the PRS (Table 5). However, in the subjects with a high vitamin C and D intake, the plasma glucose concentrations were positively linked to the PRS (Supplementary Figure S2D,E). In the group with a high coffee intake, participants with a high PRS showed a higher plasma glucose concentration than those with a low coffee intake (Table 5; Supplementary Figure S2F). Smoking had the greatest impact on the PRS interactions; the plasma glucose concentrations were much lower in nonsmokers than in former and current smokers regardless of the PRS; in former and current smokers, they were positively associated with the PRS (Table 5; Supplementary Figure S2G). Therefore, it is recommended that adults with a high PRS for the antioxidant system should increase their intake of PBD containing high levels of bioactive compounds, as well as vitamins C and D, whereas those with a low PRS could increase their coffee intake.

## 4. Discussion

Hyperglycemia increases the generation of ROS [37]. Due to the imbalance in the production of ROS and their elimination by the antioxidant system, there is an excess of ROS which can damage proteins, lipids, and nucleic acids and modulate several intracellular signaling pathways that cause insulin resistance and β-cell dysfunction [5,6]. In addition, ROS production due to hyperglycemia contributes to micro- and macrovascular diabetic complications [3]. To offset the adverse effects of ROS, stimulation of the body’s endogenous antioxidant system or intake of exogenous antioxidants can help maintain homeostasis [37]. However, a few studies have shown that genetic variants related to T2DM risk are involved in oxidative stress and the antioxidant system [38]. In the present study, we aimed to assess the association of polygenic variants related to T2DM with oxidative stress and to determine the interaction of their PRS with lifestyle factors, including nutrient intake, in a large hospital-based cohort. This study provided novel findings which suggested that the PRS of genetic variants linked to oxidative stress and the antioxidant system was positively associated with the risk of T2DM in middle-aged and elderly persons with normal body weight (average 23.9 kg/m^2^ BMI). Furthermore, the PRS interacted with DII, coffee, and vitamin D intake, as well as smoking status. Furthermore, the wild and mutated types of *GSTA5*_rs2397118 interacted with some polyphenols differently. These results suggest that the antioxidant system may play a critical role in preventing and managing T2DM, and that an exogenous antioxidant intake may help alleviate the risk of T2DM in adults genetically susceptible to oxidative stress.

The study population (Asians) had a relatively normal body weight, unlike Caucasians in a previous study [39]. Furthermore, T2DM incidence was much lower in adults with normal weight than those with obesity in Caucasians. However, its prevalence was high even in adults with normal weight, similar to obese persons [40], consistent with the present study. Although BMI was higher in the T2DM group (24.1 ± 0.14 kg/m^2^) than in the non-T2DM group (23.8 ± 0.03 kg/m^2^), the average BMI of both groups was less than 25 kg/m^2^. This is unlike the T2DM etiology in Caucasians. The primary T2DM etiology in Asians is impaired β-cell function and mass. It is explained by the genetic variants influencing susceptibility to T2DM, which vary among different ethnic groups. In Asians, genetic variants, such as the CDK5 regulatory subunit-associated protein 1 like 1 *(*CDKAL1)*_ rs35612982,* cyclin-dependent kinase inhibitor 2A/B *(*CDKN2A/B)*_rs10811661*, potassium voltage-gated channel subfamily Q member 1 *(*KCNQ1)*_rs60808706*, and GLIS family zinc finger 3 *(*GLIS3)*_rs7034200*, are mainly related to insulin production and its regulation and β-cell mass [9]. Their PRS is associated with a 5.8-fold higher risk of T2DM [9]. The PRS related to β-cell function strongly contributes to T2DM and gestational diabetes risk, particularly in Asians [9,41,42]. Some genetic variants related to inflammation and insulin resistance are more prevalent in Caucasians [43]. Therefore, genetic variants can reveal the potential etiology of T2DM in different ethnicities. The impaired β-cell function may be involved in oxidative stress.

An imbalance in endogenous and exogenous antioxidants and ROS production causes oxidative stress. However, there are few studies on the association of oxidative stress-related genetic variants with T2DM [16,44], although some genes related to oxidative stress and the antioxidant system are reported to be linked to the risk of T2DM and its complications. Interestingly, the PRS of the genetic variants related to oxidative stress was not linearly related to plasma glucose concentrations, and adjusted ORs for the PRS were lower in the oxidative stress than an antioxidant system for T2DM risk, although the genes related to oxidative stress had a higher relevance score than those related to the antioxidant system. This might have been due to the greater genetic impact of the antioxidant system in T2DM in the Korean cohort. The results suggested that ROS elimination may have protective activity against T2DM, but genetic variants related to progressed oxidative stress might be less involved in T2DM risk. T2DM patients have lower glutathione levels due to a slower rate of biosynthesis and the reduction reaction of oxidized GSH (GSSG) than non-T2DM adults [13]. The GO and KEGG analyses for oxidative stress related to T2DM did not include ROS production. However, they mainly covered cellular oxidant detoxification, response to oxidative stress, and glutathione metabolism, suggesting that genetic variants related to T2DM were mainly involved in the antioxidant system included in the present study.

Several genetic association studies have indicated that SNPs, mainly in the genes for redox state-regulating enzymes, are associated with T2DM susceptibility [45]. The SNPs of ROS production-related genes may also be linked to T2DM. ROS production mainly involves NADPH oxidase (*NOX*) composed of cytochrome b-245 light chain (*CYBA),* its heavy chain (*CYBB*), the neutrophil cytosolic factor 1 (*NCF*)-1, *NCF2*, *NCF4*, and small GTPases (*RAC1/RAC2*) [46]. Their genetic variants include *RAC1*_rs7784465, *CYBA*_rs4673, *CYBB*_rs5963327, rs6610650, and *NCF2*_rs17849502, which are reported to be associated with T2DM. However, the genes related to ROS production and their genetic variants were not significantly linked to T2DM risk from the GO and KEGG analyses in the present study. The genetic results indicated that the genetic variants related to oxidative stress were related to the antioxidant system but not ROS production and ROS-related cellular damage. The response to oxidative stress is involved in counteracting and mitigating the damaging effects of ROS, including an antioxidant system. In this study, we propose that the development of T2DM may be modulated by the contents of ROS, influenced by genes associated with the antioxidant system rather than ROS production and post-damage of the tissues, mainly pancreatic β-cells to decrease β-cell mass and function in the adults with normal weight. Specifically, the capacity of the antioxidant system to remove ROS is of primary importance in mitigating oxidative stress and influencing T2DM risk. Consequently, adults with a PRS for T2DM exhibit a lower antioxidant system capacity, leading to increased oxidative stress and a higher risk of T2DM. Therefore, individuals with a high PRS may potentially mitigate and reduce their risk of T2DM by incorporating exogenous antioxidants to enhance ROS removal.

The genetic research conducted thus far has predominantly focused on antioxidant defense enzymes, such as glutathione S-transferases (*GST*) M1, *GSTT1*, and *GSTP1*, which play a role in the conjugation of reduced glutathione with xenobiotic compounds [45]. Loss-of-function variants in these enzymes, such as GSS (rs13041792) and GGT7 (rs6119534 and rs11546155), have been associated with an increased risk of T2DM by the folding of proinsulin, which triggers an unfolded protein response [44]. This is associated with endogenous glutathione deficiency in the risk alleles. The present study also revealed significant associations between T2DM and genetic variants in *PRDX6*, *GPX1*, *GPX3*, *GSTA5*, *GCLC*, *GSR*, and *GGT1*, all of which are critical components involved in glutathione metabolism within the antioxidant system. Several studies have identified specific polymorphisms in antioxidant enzymes that are linked to T2DM risk, including homozygote deletion of *GSTM1*, heterozygote deletion of *GSTT1*, *GSTP1*_rs1695 and rs1138272, *GCLC*_rs12524494, glutamate–cysteine ligase (*GCLM*)_rs3827715 and rs41303970, glutathione synthetase (*GSS*)_rs13041792, *GSR*_rs2551715, *GGT7*_rs11546155 and rs6119534, gamma-glutamyl cyclotransferase (*GGCT*)_rs4270, *GPX1*_rs1050450, *GPX2*_rs4902346, catalase (*CAT*)_rs769217, *SOD1*_rs2234694, *SOD2*_rs4880, and *SOD3*_rs2536512 [13]. Additionally, the selenium-rich plasma protein gene *SELENOP*, primarily produced in the liver, has been shown to impair insulin signaling and β-cell function when present in excess [46]. While *NFE2L1* is not directly associated with insulin signaling and oxidative stress, it inhibits AMPK activity, thus contributing to the etiology of T2DM [47]. The activation of AMPK signaling by metformin involves suppressing NFE2L1 activity [48]. Therefore, the genes are mainly involved in T2DM risk directly or indirectly through antioxidant systems and oxidative stress, particularly glutathione metabolism-linked genes.

Although the genes found in the present study have been reported to be linked to T2DM risk in earlier studies [16,18], their genetic variants were not reported. Since many genetic variants are closely linked to each other, showing high LD (D’ > 0.3), their genetic variants might be comparable to those reported in earlier studies [14,16]. The PRS generated by summing the risk alleles of the selected genetic variants in the allelic genetic model was positively associated 1.423 times with T2DM (*p* < 0.0001), although each SNP had a low beta score and high *p*-value (*p* = 0.0017–0.0037). The PRS made from the genetic variants related to the response to oxidative stress in an allelic genetic model showed a positive association. However, the PRS for the response to oxidative stress was not linearly positively associated with plasma glucose concentrations, unlike PRS for the antioxidant system. Thus, the present study considered the PRS linked to the antioxidant system as a genetic factor linked to T2DM. Unlike the allelic genetic model, the PRS for the recessive and dominant genetic models showed less genetic impact than that for the allelic genetic model in the present study. This suggests that the risk allele may not exhibit a sufficient effect on T2DM risk in a dominant mode of action. A dose-dependent relationship existed between the number of risk alleles and the increased risk or impact on T2DM development. Individuals carrying more risk alleles, even if it was just one additional allele, were more likely to exhibit a higher risk or manifestation of the T2DM risk.

Genetic interaction with lifestyle factors can be linked to T2DM risk. However, few studies have found that genetic variants related to antioxidant systems interact with lifestyle factors influencing T2DM risk [46,47]. The interaction between PRS and dietary antioxidants influencing T2DM risk was determined since the PRS in this study was related to the antioxidant system. Interestingly, the PRS showed an interaction with oxidative stress-related nutrients such as bioactive compounds, foods with high DII, vitamin C, vitamin D, and coffee. Moreover, the PRS interacted with smoking status to affect the T2DM risk. This suggests that PRS modulated oxidative stress by altering the antioxidant system, and the increased oxidative stress was positively linked to the T2DM risk. However, plasma concentrations of antioxidants, including vitamins A, C, E, and D, were lower in T2DM patients than the non-T2DM adults [49]. Antioxidant and herbal medications are beneficial for T2DM therapy, and they elevate antioxidant enzymes such as SOD, glutathione peroxidase, and catalase in the body [50]. However, the effects of these treatments vary in individuals, which genetic differences can explain. In addition, *GSTA5*_rs2397118 is a missense mutation, and both wild and mutated types had low binding energy to some anthocyanins and flavonoids. However, wildtype and mutated *GSTA5*_rs2397118 had different binding energy to these. The low binding energy with flavonoids and anthocyanins suggested improved GSTA5 activity. Adults with high PRS need higher exogenous antioxidants to offset the increase in oxidative stress due to lower antioxidant system activity. Therefore, the results of the present study can be applied to provide precision nutrition to prevent T2DM induced by oxidative stress.

The present study revealed a novel finding that dietary antioxidants interact with the PRS linked to the antioxidant system to modulate T2DM risk in a large cohort (*n* = 58,701), which was a well-designed and well-controlled study conducted by the Korea Disease Control and Prevention Agency. However, the present study had some limitations: First, this was a cross-sectional study; therefore, the results cannot be used to draw any conclusions about cause and effect. Second, an SQFFQ was used to estimate the daily food intake of subjects over the past 6 months and included 106 food items and dishes commonly consumed by Koreans. Although the SQFFQ did not evaluate the exact amounts of the food and dishes, it adequately represented the usual nutrient intake.

In conclusion, this study demonstrated that the PRS related to the antioxidant system is associated with T2DM risk. Moreover, there was a potential indication that the intake of exogenous antioxidants could mitigate this risk in adults with a genetic predisposition to oxidative stress. Additionally, a missense mutation in the *GSTA5* gene needs further investigation regarding the polyphenol effects on T2DM risk. The results of this study could help formulate future preventive strategies and provide a basis for personalized nutrition interventions for those at risk of T2DM.

## Figures and Tables

**Table 1:** Demographic, anthropometric, biochemical, and dietary characteristics according to type 2 diabetes (T2DM) status.

Parameters	Non-T2DM (*n* = 52,634)	T2DM (*n* = 5310)
Age (years)	53.9 ± 0.03	57.2 ± 0.14 ***
Gender (men; N, %)	17,405 (33.1)	2596 (48.9)
Education (N, %)		
≤Middle school	7354 (19.0)	1137 (27.0) ***^, 1^
High school	28,463 (73.6)	2842 (67.4)
≥College	2834 (7.33)	239 (5.67)
BMI (kg/m^2^)	23.8 ± 0.02	24.7 ± 0.05 ***
Waist circumference (cm)	80.5 ± 0.03	81.5 ± 0.09 ***
Plasma glucose (mg/dL)	91.4 ± 0.09	130.8 ± 0.28 ***
HbA1c (%)	5.5 ± 0.00	7.1 ± 0.01 ***
HOMA-IR ^2^ (%)	1456 (2.77)	3098 (58.3) ***
Hs-CRP (mg/dL)	0.13 ± 0.00	0.17 ± 0.01 ***
Cardiovascular disease (N, %)	1789 (3.40)	499 (9.41) ***
Myocardial infarction (N, %)	1288 (2.45)	366 (6.90) ***
Stroke (N, %)	533 (1.01)	149 (2.81) ***
Energy intake (EER %)	97.5 ± 0.19	94.3 ± 0.57 ***
CHO (En%)	71.7 ± 0.04	71.8 ± 0.13
Fat (En%)	13.9 ± 0.03	13.6 ± 0.1 **
Protein (En%)	13.5 ± 0.02	13.6 ± 0.05
Korean balanced diet (N, %)	17,780 (33.8)	1808 (34.1)
Plant-based diet (N, %)	39,242 (74.6)	3925 (73.9)
Western-style diet (N, %)	21,576 (41.0)	1976 (37.2) ***
Rice main diet (N, %)	18,137 (34.5)	1433 (27.0) ***
Dietary inflammatory index	−20.8 ± 0.09	−21.5 ± 0.27 ***
Glycemic index	47.8 ± 0.06	46.8 ± 0.17 ***
Glycemic load	149.6 ± 0.2	146.2 ± 0.62 ***
Bioactive compounds ^3^ (mg/day)	40.5 ± 0.19	38.2 ± 0.56 **
Vitamin C (mg/day)	110.2 ± 0.35	107.6 ± 1.09 *
Sulfur microbial diet	−52.5 ± 0.44	−52.5 ± 1.34
Alcohol (g/day)	16.9 ± 0.29	16.8 ± 0.91
Coffee (cup/day)	0.71 ± 0.00	0.65 ± 0.01 ***
Exercise ^4^ (Y, %)	28,539 (54.3)	3071 (57.9) ***
Non-smoking (N, %)	48,594 (92.4)	4770 (89.9)
Former smoking (N, %)	2360 (4.49)	310 (5.84)
Smoking (N, %)	1662 (3.16)	228 (4.30) ***

Values represent the means ± standard error. ^1^ Statistical analysis between education level and T2DM status. ^2^ A 2.32 cutoff representing insulin resistance. ^3^ Bioactive compound intake was calculated by adding quercetin, luteolin, genistein, daidzein, and cyanidin intake ^4^ Defined as over 150 min per week of moderate-intensity physical activity such as brisk walking, water aerobics, bike riding, dancing, doubles tennis, pushing a lawn mower, hiking, and rollerblading. BMI, body mass index; HOMA-IR, homeostatic model assessment for insulin resistance; CRP, C-reactive protein; EER, estimated energy requirement; CHO, carbohydrates; En%, percentage based on energy intake. * Significantly different from the non-T2DM group at *p* < 0.05, ** at *p* < 0.01, and *** at *p* < 0.001.

**Table 2:** The relevance score of oxidative stress-related genes in type 2 diabetes (T2DM), oxidative stress, and antioxidant genes in the Gene Cards website.

	Gene Accession Number	Relevance Score		
		T2DM	Oxidative Stress	Antioxidant System
*GPX1*	NM_000581.4	32.39	22.18	7.99
*GSR*	NM_000637.5	30.00	28.39	15.13
*APOE*	NM_000041.4	29.95	9.21	5.25
*PON2*	NM_000305.3	22.85	11.41	5.46
*GGT1*	NM_001288833.2	22.66	9.58	3.79
*GPX3*	NM_001329790.2	21.09	11.56	4.54
*SELENOP*	NM_001085486.3	15.07	7.08	4.55
*PRDX6*	NM_004905.3	14.41	14.12	12.48
*GCLC*	NM_001197115.2	12.91	11.52	3.52
*NFE2L1*	NM_001330261.2	9.77	8.69	6.30
*GSTA5*	NM_153699.3	7.52	7.04	3.11

**Table 3:** Characteristics of genetic variants selected for antioxidant system or response to oxidative stress in the genetic variants associated with type 2 diabetes.

A. Genetic Variants Selected for the Antioxidant System											
Chr ^1^	SNP ^2^	Position	Mi ^3^	Ma ^4^	OR ^5^	SE ^6^	P ^7^	Gene Names	Location	MAF ^8^	*p*-Value for HWE ^9^
1	rs150751487	173484177	C	T	0.944	0.0193	0.0022	*PRDX6*	Intron variant	0.284	0.490
3	rs1050614	49394636	C	T	0.921	0.0407	0.0038	*GPX1*	3′ UTR variant	0.060	0.768
5	rs8177426	151023379	A	G	0.877	0.0692	0.0024	*GPX3*	Intron variant	0.028	0.822
6	rs7739421	52697404	T	C	1.070	0.0267	0.0038	*GSTA5*	Intron variant	0.132	0.957
6	rs2397118	52701143	C	T	0.942	0.0274	0.0020	*GSTA5*	Missense variant P.Val(C)55Leu(T)	0.157	0.236
6	rs74515451	53436825	C	A	1.110	0.0282	0.0067	*GCLC*	Intron variant	0.109	0.210
6	rs78386169	53474246	G	A	0.874	0.0467	0.0011	*GCLC*	Intron variant	0.053	0.136
7	rs10274638	114914505	G	A	1.092	0.0316	0.0019	*GSR*	Intron variant	0.095	0.205
22	rs2076999	25003934	A	G	1.058	0.0186	0.0017	*GGT1*	3′ UTR variant	0.351	0.731
B. Genetic Variants Selected for the Response to Oxidative Stress											
Chr ^1^	SNP ^2^	Position	Mi ^3^	Ma ^4^	OR ^5^	SE ^6^	P ^7^	Gene names	Location	MAF ^8^	*p*-Value for HWE ^9^
1	rs150751487	173484177	C	T	0.944	0.0193	0.00215	*PRDX6*	Intron variant	0.284	0.490
3	rs1050614	49394636	C	T	0.921	0.0407	0.00376	*GPX1*	3′ UTR variant	0.060	0.768
5	rs28919269	42804538	G	C	1.058	0.0199	0.00224	*SELENOP*	Intron variant	0.287	0.226
5	rs8177426	151023379	A	G	0.877	0.0652	0.00243	*GPX3*	Intron variant	0.028	0.822
6	rs74515451	53436825	C	A	1.110	0.0302	0.00671	*GCLC*	Intron variant	0.109	0.2100
7	rs6462738	37235191	C	T	1.077	0.0234	0.00300	*PON2*	Intron variant	0.121	0.877
17	rs182345537	46128778	T	G	1.222	0.0809	0.00386	*NFE2L1*	Missense variant	0.012	0.730
19	rs769450	45410444	A	G	0.940	0.0268	0.00288	*APOE*	Intron variant	0.205	0.630

^1^ Chromosome. ^2^ Single-nucleotide polymorphism. ^3^ Minor allele. ^4^ Major allele. ^5^ Odds ratio. ^6^ Standard error. ^7^ *p*-Value for OR adjusting for age, gender, residence area, survey year, body mass index, daily energy intake, education, and income in the city cohort. ^8^ Minor allele frequency. ^9^ Hardy–Weinberg equilibrium.

**Table 4:** Biding energy of anthocyanins and flavonoids to the active site of the GSTA5 proteins (Val55Leu) converted from the C and T alleles of rs2397118.

Food Components	Valine at 55	Leucine at 55	Food Components	Valine at 55	Leucine at 55
Diosgenin 3-[glucosyl-(1->4)-[glucopyranosyl-(1->6)]-glucopyranosyl-(1->4)-rhamnosyl-(1->4)-[rhamnosyl-(1->2)]-glucoside]	−11.8	−11.8	(Cyanidin 3-O-(3-O-acetyl-beta-glucoside) (kaempferol 3-O-(2-O-beta-glucosyl-beta-glucoside)-7-O-beta-glucosiduronic acid) malonate	−9.9	−10.7
Matesaponin 4	−11.3	−11.4	Lupeoside	−10.8	−10.8
Malvidin 3-chlorogenic acid glucoside	−9.3	−11.2	Kaempferol 3-[4″-(*p*-coumaroylglucosyl)rhamnoside]	−10.7	−10.8
Azaspiracid 5	−10.7	−11.2	Delphinidin 3-[6″-(4″′-p-coumaroylrhamnosyl)glucoside] 5-glucoside	−10.0	−10.7
Theadibenzotropolone A	−11.9	−11.2	Theaflavin 3-gallate	−10.8	−10.8
Vitilagin	−11.2	−11.1	Kaempferol 3-(p-coumaroyl-glucoside)	−10.8	−10.7
Isotheaflavin 3′-gallate	−11.1	−11	Kuwanon Z	−10.6	−10.7
Isovitexin 2′′-O-(6′′′-feruloyl)glucoside	−11.0	−11	Fistuloside A	−10.7	−10.7
Cyanidin 3-O-(2′′-xylosyl-6′′-(6′′-p-coumaroyl-glucosyl)-galactoside)	−10.4	−10.9	Kaempferol 3-[2″-(p-coumaroylglucosyl)rhamnoside]	−10.6	−10.7
Malvidin 3-caffeoyl-glucoside	−10.9	−10.9	Kaempferol 3-rhamnosyl-(1->3)-rhamnosyl-(1->6)-glucoside	−10.5	−10.6
Quercetin 3-(6″-p-coumarylsophorotrioside)	−10.8	−10.8	Pelargonidin 3-O-[2-O-(6-(E)-feruloyl-beta-D-glucopyranosyl)-6-O-(E)-p-coumaroyl-beta-D-glucopyranoside] 5-O-(beta-D-glucopyranoside)	−10.6	−10.6
Delphinidin 3-caffeoylglucoside	−10.7	−10.8	Asterlingulatoside D	−9.4	−11.3
Petunidin 3-(4″-p-coumaroyl-rutinoside)	−10.8	−10.8	Kaempferol 3-O-rhamnosyl-rhamnosyl-glucoside	−8.7	−10.4
Cyanidin 3-dicaffeoyl-sophoroside 5-glucoside	−10.2	−10.8	(Cyanidin 3-O-beta-glucoside)(kaempferol 3-O-(2-O-beta-glucosyl-beta-glucoside)-7-O-beta-glucosiduronic acid) malonate	−8.9	−11.1
β-Chlorogenin 3-[2″,4″-dirhamnosylglucoside]	−10.7	−10.8			

**Table 5:** Adjusted odds ratios for the type 2 diabetes (T2DM) risk by polygenetic risk scores of the antioxidant system after covariate adjustments according to dietary antioxidant intake.

	Low PRS	Medium PRS	High PRS	Gene–Nutrient Interaction *p*-Value
Low PBD ^1^ High PBD	1 1	1.172 (1.001–1.373) 1.189 (1.040–1.359)	1.378 (1.206–1.575) 1.423 (1.220–1.660)	0.0705
Low DII ^1^ High DII	1 1	1.156 (1.033–1.294) 1.385 (1.109–1.729)	1.292 (1.135–1.472) 1.900 (1.486–2.431)	0.0303
Low bioactive ^1^ High bioactive	1 1	1.236 (1.053–1.450) 1.075 (0.841–1.374)	1.518 (1.265–1.822) 1.208 (0.906–1.611)	0.0444
Low vitamin C ^2^ High vitamin C	1 1	1.138 (0.937–1.379) 1.229 (1.020–1.485)	1.266 (1.113–1.678) 1.653 (1.315–2.076)	0.0342
Low vitamin D ^2^ High vitamin D	1 1	1.056 (0.806–1.383) 1.233 (1.056–1.439)	1.207 (0.884–1.648) 1.500 (1.256–1.791)	0.0453
Low coffee ^1^ High coffee	1 1	1.027 (0.840–1.256) 1.320 (1.103–1.579)	1.314 (1.044–1.653) 1.492 (1.212–1.837)	0.037
Non-smoking + former smoking Smoking	1 1	1.138 (0.958–1.352) 1.289 (1.042–1.594)	1.360 (1.116–1.656) 1.546 (1.209–1.976)	0.0186

Values represent adjusted odd ratios and 95% confidence intervals. Covariates included age, sex, education, income, energy intake (percentage of estimated energy requirement), residence areas, daily activity, alcohol intake, and smoking status. The PRS with eight SNPs of the best GMDR model was divided into three categories according to the number of the risk alleles: ≤3, 4–5, and ≥6 risk alleles in the PRS into low PRS, medium PRS, and high PRS, respectively. The reference was low PRS. PBD, plant-based diet; DII, dietary inflammatory index. Bioactive compounds were calculated by summing quercetin, luteolin, genistein, daidzein, and cyanidin intake. ^1^ Lower than 25th percentiles. ^2^ Lower than dietary reference index (100 mg/day for vitamin C and 10 µg/day for vitamin D).

## Data Availability

The data were deposited in the Korean biobank (Osong, Republic of Korea).

## Supplementary Materials

The following supporting information can be downloaded at https://www.mdpi.com/article/10.3390/antiox12061280/s1, Figure S1. Association of polygenic risk scores (PRS) with type 2 diabetes risk in the dominant (DGM) and recessive genetic models (RGM); Figure S2. Adjusted means and standard errors of fasting plasma glucose concentrations of participants with low, medium, or high polygenic risk scores (PRS) determined using the antioxidant system-related gene model. Table S1. Relative scores of genes related to oxidative stress.
