# Supplementary material for: Polygenic Variants Linked to Oxidative Stress and the Antioxidant System Are Associated with Type 2 Diabetes Risk and Interact with Lifestyle Factors
Source: Antioxidants (Basel). 2023 Jun 15;12(6):1280. doi: 10.3390/antiox12061280 (PMC10295348; doi:10.3390/antiox12061280)
Supplement: Supplementary file 1 [file antioxidants-12-01280-s001.zip › antioxidants-2413847-supplementary.pdf]

Supplementary Table S1. Relevance scores of genes related to oxidative stress.

Gene Symbol	Description	Category	Gifts	GC Id	Relevance score
SOD2-OT1	SOD2 Overlapping Transcript 1	RNA Gene	15	GC06M159772	47.87
CAT	Catalase	Protein Coding	59	GC11P034460	41.88
SOD1	Superoxide Dismutase 1	Protein Coding	60	GC21P031659	40.59
NFE2L2	NFE2 Like BZIP Transcription Factor 2	Protein Coding	58	GC02M177227	39.82
OXSR1	Oxidative Stress Responsive Kinase 1	Protein Coding	48	GC03P038183	36.17
OSGIN1	Oxidative Stress Induced Growth Inhibitor 1	Protein Coding	42	GC16P083934	33.88
SOD2	Superoxide Dismutase 2	Protein Coding	54	GC06M159669	32.69
OSER1	Oxidative Stress Responsive Serine Rich 1	Protein Coding	39	GC20M044195	32.06
TP53	Tumor Protein P53	Protein Coding	61	GC17M007661	29.4
OSGIN2	Oxidative Stress Induced Growth Inhibitor Family Member 2	Protein Coding	38	GC08P089901	29.1
GSR	Glutathione-Disulfide Reductase	Protein Coding	56	GC08M030678	28.39
HMOX1	Heme Oxygenase 1	Protein Coding	59	GC22P035380	27.88
PARK7	Parkinsonism Associated Deglycase	Protein Coding	54	GC01P008181	24.97
MAPK8	Mitogen-Activated Protein Kinase 8	Protein Coding	57	GC10P048306	23.53
SIRT1	Sirtuin 1	Protein Coding	56	GC10P067884	22.91
BDNF-AS	BDNF Antisense RNA	RNA Gene	24	GC11P027578	22.88
TXN	Thioredoxin	Protein Coding	52	GC09M110243	22.45

NQO1	NAD(P)H Quinone Dehydrogenase 1	Protein Coding	55	GC16M069706	22.4
GPX1	Glutathione Peroxidase 1	Protein Coding	52	GC03M052166	22.18
MAP3K5	Mitogen-Activated Protein Kinase Kinase Kinase 5	Protein Coding	55	GC06M136557	21.06
FOXO3	Forkhead Box O3	Protein Coding	53	GC06P108559	20.88
FOXO1	Forkhead Box O1	Protein Coding	57	GC13M040555	20.32
XDH	Xanthine Dehydrogenase	Protein Coding	54	GC02M031334	19.89
LINC01672	Long Intergenic Non-Protein Coding RNA 1672	RNA Gene	14	GC01P007053	19.73
MAPK14	Mitogen-Activated Protein Kinase 14	Protein Coding	58	GC06P100333	19.6
CASP3	Caspase 3	Protein Coding	56	GC04M184627	19.28
KEAP1	Kelch Like ECH Associated Protein 1	Protein Coding	56	GC19M010486	19.23
GSTM1	Glutathione S-Transferase Mu 1	Protein Coding	49	GC01P109687	18.83
PON1	Paraoxonase 1	Protein Coding	54	GC07M095297	18.74
MAPK1	Mitogen-Activated Protein Kinase 1	Protein Coding	60	GC22M021759	18.44
TNF	Tumor Necrosis Factor	Protein Coding	59	GC06P100202	18.15
PRDX2	Peroxiredoxin 2	Protein Coding	53	GC19M012796	18.03
MPO	Myeloperoxidase	Protein Coding	60	GC17M058269	17.75
JUN	Jun Proto-Oncogene, AP-1 Transcription Factor Subunit	Protein Coding	55	GC01M058780	17.46
IL6	Interleukin 6	Protein Coding	59	GC07P022725	17.31
CYCS	Cytochrome C, Somatic	Protein Coding	55	GC07M025118	17.16

PARP1	Poly(ADP-Ribose) Polymerase 1	Protein Coding	58	GC01M226360	16.93
TMX2-CTNND1	TMX2-CTNND1 Readthrough (NMD Candidate)	RNA Gene	19	GC11P057712	16.74
APP	Amyloid Beta Precursor Protein	Protein Coding	58	GC21M025880	16.56
CYBA	Cytochrome B-245 Alpha Chain	Protein Coding	55	GC16M088643	16.32
SOD3	Superoxide Dismutase 3	Protein Coding	50	GC04P024798	16.19
TRPM2	Transient Receptor Potential Cation Channel Subfamily M Member 2	Protein Coding	48	GC21P044350	16.17
GSTP1	Glutathione S-Transferase Pi 1	Protein Coding	57	GC11P067583	15.83
NOS3	Nitric Oxide Synthase 3	Protein Coding	56	GC07P151739	15.59
CERNA3	Competing Endogenous LncRNA 3 For MiR-645	RNA Gene	14	GC08P056085	15.34
PGR-AS1	PGR Antisense RNA 1	RNA Gene	13	GC11P101131	15.15
PRDX3	Peroxiredoxin 3	Protein Coding	52	GC10M119167	15.14
NOS2	Nitric Oxide Synthase 2	Protein Coding	56	GC17M027756	15
GSTT1	Glutathione S-Transferase Theta 1	Protein Coding	38	GC22Mi00270	14.92
SMAD5-AS1	SMAD5 Antisense RNA 1	RNA Gene	26	GC05M136129	14.75
OGG1	8-Oxoguanine DNA Glycosylase	Protein Coding	54	GC03P014247	14.63
PRDX5	Peroxiredoxin 5	Protein Coding	51	GC11P064532	14.55
CRP	C-Reactive Protein	Protein Coding	53	GC01M159731	14.33
INS	Insulin	Protein Coding	54	GC11M002159	14.3
G6PD	Glucose-6-Phosphate Dehydrogenase	Protein Coding	57	GC0XM154573	14.3
TXNIP	Thioredoxin Interacting Protein	Protein Coding	46	GC01M145992	14.15

PRDX6	Peroxiredoxin 6	Protein Coding	53	GC01P173477	14.12
ABL1	ABL Proto-Oncogene 1, Non-Receptor Tyrosine Kinase	Protein Coding	59	GC09P130713	13.82
AKT1	AKT Serine/Threonine Kinase 1	Protein Coding	61	GC14M104769	13.67
PRKCD	Protein Kinase C Delta	Protein Coding	59	GC03P053156	13.61
BAX	BCL2 Associated X, Apoptosis Regulator	Protein Coding	57	GC19P048954	13.57
SHC1	SHC Adaptor Protein 1	Protein Coding	51	GC01M154962	13.45
BCL2	BCL2 Apoptosis Regulator	Protein Coding	57	GC18M063123	13.33
PRKN	Parkin RBR E3 Ubiquitin Protein Ligase	Protein Coding	55	GC06M161348	13.22
TXN2	Thioredoxin 2	Protein Coding	50	GC22M036467	13.08
PRDX1	Peroxiredoxin 1	Protein Coding	56	GC01M045590	13.08
SNCA	Synuclein Alpha	Protein Coding	59	GC04M089724	13.05
TXNRD1	Thioredoxin Reductase 1	Protein Coding	51	GC12P104215	13.02
GPX7	Glutathione Peroxidase 7	Protein Coding	49	GC01P052602	12.96
NOX4	NADPH Oxidase 4	Protein Coding	50	GC11M089324	12.87
UCP2	Uncoupling Protein 2	Protein Coding	53	GC11M073974	12.82
CYBB	Cytochrome B-245 Beta Chain	Protein Coding	56	GC0XP037780	12.79
CXCL8	C-X-C Motif Chemokine Ligand 8	Protein Coding	51	GC04P073740	12.79
H19	H19 Imprinted Maternally Expressed Transcript	RNA Gene	30	GC11M001995	12.69
HSPA4	Heat Shock Protein Family A (Hsp70) Member 4	Protein Coding	50	GC05P133054	12.55

ALB	Albumin	Protein Coding	56	GC04P073397	12.53
APEX1	Apurinic/Apyrimidinic Endodeoxyribonuclease 1	Protein Coding	52	GC14P020455	12.47
CP	Ceruloplasmin	Protein Coding	55	GC03M149162	12.15
MSRA	Methionine Sulfoxide Reductase A	Protein Coding	48	GC08P010054	12.12
HSPB1	Heat Shock Protein Family B (Small) Member 1	Protein Coding	59	GC07P076302	11.94
SP1	Sp1 Transcription Factor	Protein Coding	52	GC12P053380	11.93
NFKB1	Nuclear Factor Kappa B Subunit 1	Protein Coding	60	GC04P102501	11.85
PRKD1	Protein Kinase D1	Protein Coding	56	GC14M029576	11.71
SQSTM1	Sequestosome 1	Protein Coding	56	GC05P179806	11.59
GPX3	Glutathione Peroxidase 3	Protein Coding	48	GC05P150997	11.56
GCLC	Glutamate-Cysteine Ligase Catalytic Subunit	Protein Coding	52	GC06M053497	11.52
PINK1	PTEN Induced Kinase 1	Protein Coding	53	GC01P020634	11.44
PON2	Paraoxonase 2	Protein Coding	50	GC07M095404	11.41
ADIPOQ	Adiponectin, C1Q And Collagen Domain Containing	Protein Coding	53	GC03P186842	11.4
TGFB1	Transforming Growth Factor Beta 1	Protein Coding	60	GC19M041301	11.4
MIR7-3HG	MIR7-3 Host Gene	RNA Gene	29	GC19P005386	11.38
MAPK10	Mitogen-Activated Protein Kinase 10	Protein Coding	56	GC04M085990	11.26
PRDX4	Peroxiredoxin 4	Protein Coding	48	GC0XP023665	11.19
MAPK3	Mitogen-Activated Protein Kinase 3	Protein Coding	56	GC16M040043	11.02
NOX1	NADPH Oxidase 1	Protein Coding	50	GC0XM100843	11.02

FOS	Fos Proto-Oncogene, AP-1 Transcription Factor Subunit	Protein Coding	57	GC14P075278	10.87
ATF4	Activating Transcription Factor 4	Protein Coding	52	GC22P039519	10.71
MSRB2	Methionine Sulfoxide Reductase B2	Protein Coding	46	GC10P023095	10.65
GAPDH	Glyceraldehyde-3-Phosphate Dehydrogenase	Protein Coding	57	GC12P026338	10.58
BCL2L1	BCL2 Like 1	Protein Coding	54	GC20M031664	10.44
SRXN1	Sulfiredoxin 1	Protein Coding	41	GC20M000647	10.42
CCL2	C-C Motif Chemokine Ligand 2	Protein Coding	56	GC17P034255	10.39
NOS1	Nitric Oxide Synthase 1	Protein Coding	56	GC12M117208	10.24
CYP1A1	Cytochrome P450 Family 1 Subfamily A Member 1	Protein Coding	55	GC15M074719	10.14
HIF1A	Hypoxia Inducible Factor 1 Subunit Alpha	Protein Coding	55	GC14P061695	10.13
SRC	SRC Proto-Oncogene, Non-Receptor Tyrosine Kinase	Protein Coding	58	GC20P037344	10.13
SIRT3	Sirtuin 3	Protein Coding	54	GC11M000215	10.12
AGER	Advanced Glycosylation End-Product Specific Receptor	Protein Coding	54	GC06M032180	10.07
CYP2E1	Cytochrome P450 Family 2 Subfamily E Member 1	Protein Coding	52	GC10P133520	9.96
SIRT2	Sirtuin 2	Protein Coding	55	GC19M038878	9.95
SELENON	Selenoprotein N	Protein Coding	44	GC01P025800	9.93
ATM	ATM Serine/Threonine Kinase	Protein Coding	60	GC11P108222	9.91

LINC02605	Long Intergenic Non-Protein Coding RNA 2605	RNA Gene	14	GC08P078838	9.88
PPARGC1A	PPARG Coactivator 1 Alpha	Protein Coding	53	GC04M023755	9.86
ATF2	Activating Transcription Factor 2	Protein Coding	54	GC02M175072	9.82
EMSLR	E2F1 MRNA Stabilizing LncRNA	RNA Gene	15	GC07P101962	9.65
MSRB1	Methionine Sulfoxide Reductase B1	Protein Coding	45	GC16M009777	9.62
GGT1	Gamma-Glutamyltransferase 1	Protein Coding	56	GC22P024583	9.58
CYGB	Cytoglobin	Protein Coding	45	GC17M076527	9.55
ANXA5	Annexin A5	Protein Coding	53	GC04M121667	9.55
JUNB	JunB Proto-Oncogene, AP-1 Transcription Factor Subunit	Protein Coding	48	GC19P012791	9.51
CDKN2A	Cyclin Dependent Kinase Inhibitor 2A	Protein Coding	59	GC09M021967	9.46
TMEM161A	Transmembrane Protein 161A	Protein Coding	43	GC19M019120	9.43
GPX4	Glutathione Peroxidase 4	Protein Coding	53	GC19P001103	9.42
MGST1	Microsomal Glutathione S-Transferase 1	Protein Coding	50	GC12P016347	9.38
VEGFA	Vascular Endothelial Growth Factor A	Protein Coding	56	GC06P043770	9.28
HSPA1A	Heat Shock Protein Family A (Hsp70) Member 1A	Protein Coding	53	GC06P100221	9.27
PRKCB	Protein Kinase C Beta	Protein Coding	55	GC16P044702	9.25
CREB1	CAMP Responsive Element Binding Protein 1	Protein Coding	56	GC02P207529	9.22
CAV1	Caveolin 1	Protein Coding	55	GC07P116524	9.22
APOE	Apolipoprotein E	Protein Coding	57	GC19P079864	9.21

AGTR1	Angiotensin II Receptor Type 1	Protein Coding	58	GC03P148697	9.21
NUDT1	Nudix Hydrolase 1	Protein Coding	48	GC07P002242	9.18
PTGS2	Prostaglandin-Endoperoxide Synthase 2	Protein Coding	56	GC01M186671	9.13
PPARG	Peroxisome Proliferator Activated Receptor Gamma	Protein Coding	60	GC03P012287	9.11
DDIT3	DNA Damage Inducible Transcript 3	Protein Coding	52	GC12M057516	9.1
TF	Transferrin	Protein Coding	57	GC03P135833	9.06
NOL3	Nucleolar Protein 3	Protein Coding	49	GC16P067222	9.04
CASP9	Caspase 9	Protein Coding	53	GC01M015491	8.99
DHCR24	24-Dehydrocholesterol Reductase	Protein Coding	52	GC01M054849	8.96
LMNA	Lamin A/C	Protein Coding	56	GC01P156082	8.95
GLRX	Glutaredoxin	Protein Coding	50	GC05M095752	8.9
MAPKAPK2	MAPK Activated Protein Kinase 2	Protein Coding	54	GC01P206684	8.89
SLC7A11	Solute Carrier Family 7 Member 11	Protein Coding	51	GC04M138164	8.85
FXN	Frataxin	Protein Coding	52	GC09P069035	8.8
CDKN1A	Cyclin Dependent Kinase Inhibitor 1A	Protein Coding	56	GC06P100341	8.74
PLA2G7	Phospholipase A2 Group VII	Protein Coding	56	GC06M046704	8.71
NFE2L1	NFE2 Like BZIP Transcription Factor 1	Protein Coding	50	GC17P072183	8.69
ACE	Angiotensin I Converting Enzyme	Protein Coding	59	GC17P063477	8.68
PPIA	Peptidylprolyl Isomerase A	Protein Coding	52	GC07P044811	8.65
TXNRD2	Thioredoxin Reductase 2	Protein Coding	51	GC22M019863	8.65

PRKCA	Protein Kinase C Alpha	Protein Coding	57	GC17P066302	8.59
PRKCG	Protein Kinase C Gamma	Protein Coding	58	GC19P053879	8.53
MMP2	Matrix Metallopeptidase 2	Protein Coding	60	GC16P055390	8.53
SCARNA5	Small Cajal Body-Specific RNA 5	RNA Gene	22	GC02P233275	8.53
GLRX2	Glutaredoxin 2	Protein Coding	44	GC01M193065	8.41
OXR1	Oxidation Resistance 1	Protein Coding	46	GC08P106271	8.4
SIRT6	Sirtuin 6	Protein Coding	52	GC19M004174	8.35
MAP2K4	Mitogen-Activated Protein Kinase Kinase 4	Protein Coding	53	GC17P012020	8.32
TRR-TCT2-1	TRNA-Arg (Anticodon TCT) 2-1	RNA Gene	11	GC17P012440	8.29
FAM120A	Family With Sequence Similarity 120A	Protein Coding	45	GC09P094687	8.28
RPS6KA5	Ribosomal Protein S6 Kinase A5	Protein Coding	53	GC14M090847	8.23
HMGB1	High Mobility Group Box 1	Protein Coding	55	GC13M030456	8.18
JUND	JunD Proto-Oncogene, AP-1 Transcription Factor Subunit	Protein Coding	48	GC19M018279	8.16
MDM2	MDM2 Proto-Oncogene	Protein Coding	59	GC12P068808	8.12
BCL2L11	BCL2 Like 11	Protein Coding	52	GC02P111119	8.05
TERT	Telomerase Reverse Transcriptase	Protein Coding	59	GC05M001253	8.04
MAP2K6	Mitogen-Activated Protein Kinase Kinase 6	Protein Coding	53	GC17P069414	8.03
NR2C2	Nuclear Receptor Subfamily 2 Group C Member 2	Protein Coding	49	GC03P014947	8
FOXO4	Forkhead Box O4	Protein Coding	48	GC0XP071095	7.99

HSPA5	Heat Shock Protein Family A (Hsp70) Member 5	Protein Coding	55	GC09M125234	7.96
GSS	Glutathione Synthetase	Protein Coding	53	GC20M034928	7.87
PRKD2	Protein Kinase D2	Protein Coding	53	GC19M046674	7.87
STK24	Serine/Threonine Kinase 24	Protein Coding	51	GC13M098445	7.82
STAT3	Signal Transducer And Activator Of Transcription 3	Protein Coding	61	GC17M042313	7.81
MAPK9	Mitogen-Activated Protein Kinase 9	Protein Coding	56	GC05M180269	7.79
ICAM1	Intercellular Adhesion Molecule 1	Protein Coding	57	GC19P010988	7.79
KRAS	KRAS Proto-Oncogene, GTPase	Protein Coding	59	GC12M025204	7.76
LEP	Leptin	Protein Coding	53	GC07P128241	7.74
IL1B	Interleukin 1 Beta	Protein Coding	54	GC02M112829	7.73
STUB1	STIP1 Homology And U-Box Containing Protein 1	Protein Coding	53	GC16P019817	7.71
VCAM1	Vascular Cell Adhesion Molecule 1	Protein Coding	52	GC01P100719	7.69
SESN2	Sestrin 2	Protein Coding	46	GC01P029272	7.67
BACH1	BTB Domain And CNC Homolog 1	Protein Coding	48	GC21P029194	7.67
GSTM3	Glutathione S-Transferase Mu 3	Protein Coding	53	GC01M109733	7.66
EDN1	Endothelin 1	Protein Coding	55	GC06P012256	7.63
GAS5	Growth Arrest Specific 5	RNA Gene	26	GC01M173947	7.6
HSPA8	Heat Shock Protein Family A (Hsp70) Member 8	Protein Coding	56	GC11M123057	7.55
GSTA5	Glutathione S-Transferase Alpha 5	Protein Coding	43	GC06M052831	7.53

TNFAIP8L1	TNF Alpha Induced Protein 8 Like 1	Protein Coding	39	GC19P004639	7.53
MAPT	Microtubule Associated Protein Tau	Protein Coding	57	GC17P045894	7.48
SERPINE1	Serpin Family E Member 1	Protein Coding	57	GC07P101127	7.47
NRF1	Nuclear Respiratory Factor 1	Protein Coding	50	GC07P129611	7.42
BAD	BCL2 Associated Agonist Of Cell Death	Protein Coding	51	GC11M106036	7.4
NGB	Neuroglobin	Protein Coding	43	GC14M077265	7.39
GCLM	Glutamate-Cysteine Ligase Modifier Subunit	Protein Coding	48	GC01M093885	7.37
MAOA	Monoamine Oxidase A	Protein Coding	56	GC0XP043654	7.36
LRRK2	Leucine Rich Repeat Kinase 2	Protein Coding	57	GC12P040196	7.27
PPIF	Peptidylprolyl Isomerase F	Protein Coding	51	GC10P096513	7.25
CAMK2G	Calcium/Calmodulin Dependent Protein Kinase II Gamma	Protein Coding	55	GC10M073812	7.23
PSEN1	Presenilin 1	Protein Coding	60	GC14P073136	7.23
SCARA3	Scavenger Receptor Class A Member 3	Protein Coding	44	GC08P027633	7.18
RAC1	Rac Family Small GTPase 1	Protein Coding	56	GC07P006377	7.16
BRF2	BRF2 RNA Polymerase III Transcription Initiation Factor Subunit	Protein Coding	44	GC08M037820	7.15
NLRP3	NLR Family Pyrin Domain Containing 3	Protein Coding	55	GC01P247415	7.11
HBG2	Hemoglobin Subunit Gamma 2	Protein Coding	48	GC11M007502	7.09
CASP8	Caspase 8	Protein Coding	59	GC02P201233	7.08
PIK3CG	Phosphatidylinositol-4,5-Bisphosphate 3-Kinase Catalytic Subunit Gamma	Protein Coding	55	GC07P106865	7.07

FDXR	Ferredoxin Reductase	Protein Coding	51	GC17M074862	7.06
MAPKAPK3	MAPK Activated Protein Kinase 3	Protein Coding	56	GC03P050611	7.05
GSTA1	Glutathione S-Transferase Alpha 1	Protein Coding	48	GC06M052791	7.04
RAF1	Raf-1 Proto-Oncogene, Serine/Threonine Kinase	Protein Coding	61	GC03M012583	7.03

Supplementary **Figure S1**. Association of polygenic risk scores (PRS) with type 2 diabetes risk in the dominant (DGM) and recessive genetic models (RGM).

The PRS models for the antioxidant system and the response to oxidative stress associated with type 2 diabetes risk. Heterozygote was considered a risk group in DGM, while it was a non-risk group in RGM. The PRS was calculated by summing the number of risk alleles in the assigned model. PRS for the antioxidant system was similarly categorized into <5, 5, and ≥6 for DGM and <7, 7-8, ≥9 for RGM. The PRS for the response to oxidative stress was classified into the Low, Medium, and High groups, namely, <4, 4, and ≥5 for DGM and <5, 5, and ≥6 for RGM. The adjusted ORs for the PRS models were calculated by adjusting age, gender, education, income, occupation, residence area, and energy intake (percentage of estimated energy requirement) (covariates 1), variables in covariate 1 plus regular exercise, alcohol intake, and smoking status (covariates 2). Abbreviations: OR: Odds ratio; PRS: Polygenic risk score.

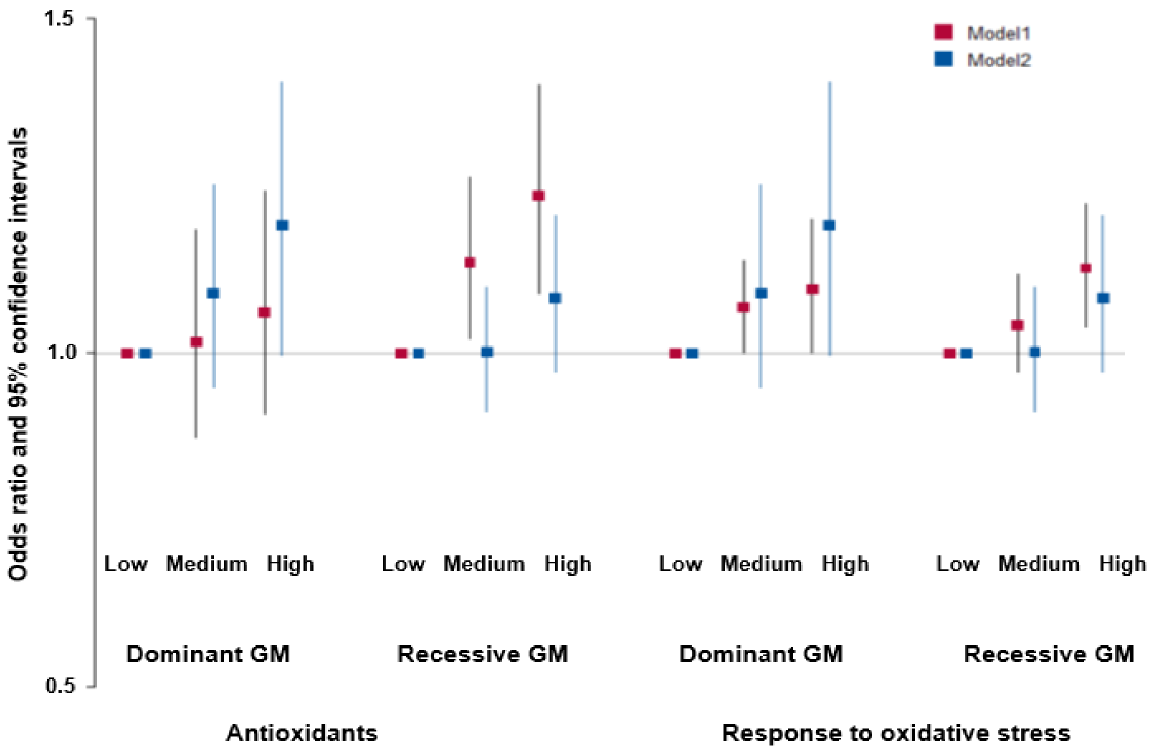

Supplementary **Figure S2.** Adjusted means and standard errors of fasting plasma glucose concentrations of participants with low, medium, or high polygenic risk scores (PRS) determined using the antioxidant system-related gene model.

A

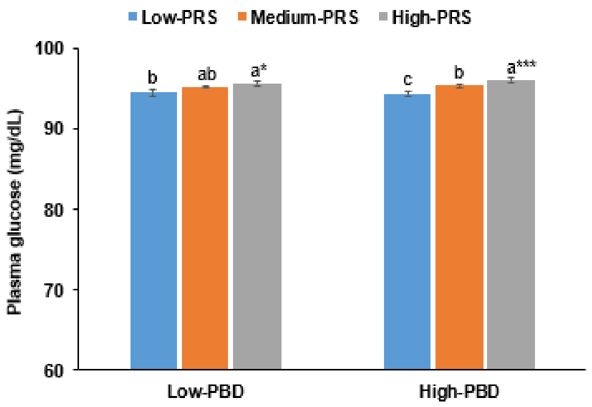

B

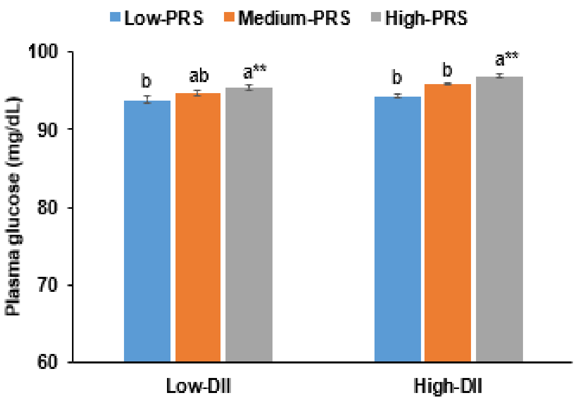

C

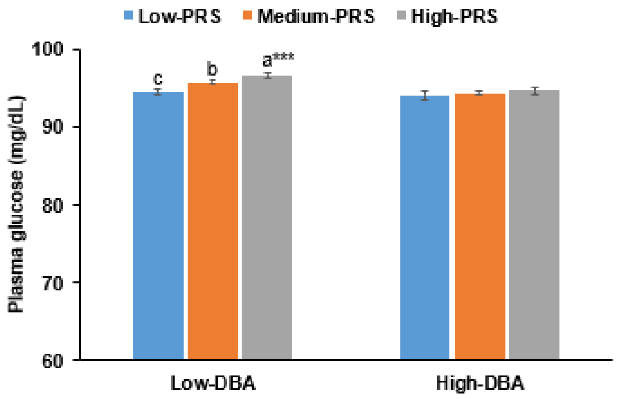

D

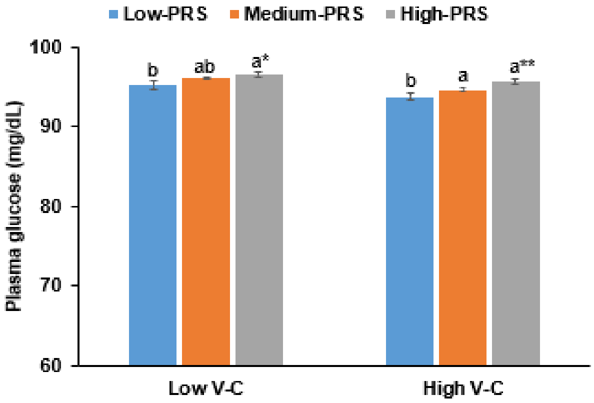

E

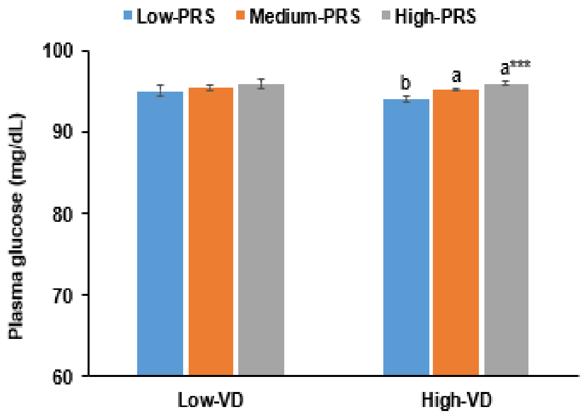

F

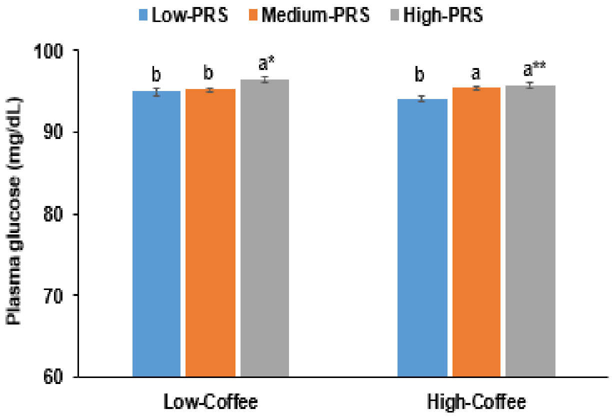

G

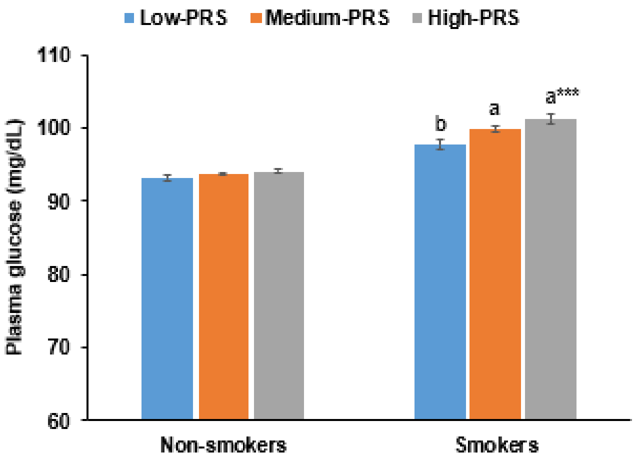

(A) Participants with the PRS categories according to plant-based diet (PBD) intake (a cutoff value: 25th percentile); **(B)** Participants with the PRS categories according to the PRS categories by dietary inflammatory index (DII) (a cutoff value: 25th percentile); **(C)** Participants with the PRS categories according to dietary bioactive compounds (DBA) (a cutoff value: 25th percentile). Dietary bioactive compounds were calculated by summing quercetin, luteolin, genistein, daidzein, and cyanidin intake; (**D)** Participants with PRS categories according to vitamin C (VC) intake (a cutoff value: 100 mg/day); **(E)** Participants with PRS categories according to vitamin D (VD) intake (a cutoff value: 10 ug/day); **(F)** Participants with PRS categories according to coffee intake (a cutoff value: the 25th percentile); **(G)** Participants with PRS categories according to smoking status.

* Significantly different among PRS groups in each category (eg. smoking status)at P<0.05, ** at P<0.01, and *** at P<0.001. a,b,c Different letters on the bars, indicate a significant difference among the PRS groups by Tukey's test at P<0.05. No alphabet indicates no significant difference among the PRS groups.
